# Control of Translation and miRNA-Dependent Repression by a Novel Poly(A) Binding Protein, hnRNP-Q

**DOI:** 10.1371/journal.pbio.1001564

**Published:** 2013-05-21

**Authors:** Yuri V. Svitkin, Akiko Yanagiya, Alexey E. Karetnikov, Tommy Alain, Marc R. Fabian, Arkady Khoutorsky, Sandra Perreault, Ivan Topisirovic, Nahum Sonenberg

**Affiliations:** 1 Department of Biochemistry and Goodman Cancer Research Centre, McGill University, Montreal, Quebec, Canada; 2 Lady Davis Institute for Medical Research, Jewish General Hospital, Department of Oncology, McGill University, Montreal, Quebec, Canada; University of California, San Diego, United States of America

## Abstract

Translation control often operates via remodeling of messenger ribonucleoprotein particles. The poly(A) binding protein (PABP) simultaneously interacts with the 3′ poly(A) tail of the mRNA and the eukaryotic translation initiation factor 4G (eIF4G) to stimulate translation. PABP also promotes miRNA-dependent deadenylation and translational repression of target mRNAs. We demonstrate that isoform 2 of the mouse heterogeneous nuclear protein Q (hnRNP-Q2/SYNCRIP) binds poly(A) by default when PABP binding is inhibited. In addition, hnRNP-Q2 competes with PABP for binding to poly(A) in vitro. Depleting hnRNP-Q2 from translation extracts stimulates cap-dependent and IRES-mediated translation that is dependent on the PABP/poly(A) complex. Adding recombinant hnRNP-Q2 to the extracts inhibited translation in a poly(A) tail-dependent manner. The displacement of PABP from the poly(A) tail by hnRNP-Q2 impaired the association of eIF4E with the 5′ m^7^G cap structure of mRNA, resulting in the inhibition of 48S and 80S ribosome initiation complex formation. In mouse fibroblasts, silencing of hnRNP-Q2 stimulated translation. In addition, hnRNP-Q2 impeded let-7a miRNA-mediated deadenylation and repression of target mRNAs, which require PABP. Thus, by competing with PABP, hnRNP-Q2 plays important roles in the regulation of global translation and miRNA-mediated repression of specific mRNAs.

## Abstract 2

The heterogeneous nuclear ribonucleoprotein Q2 competitively binds mRNA poly(A) tails to regulate translational and miRNA-related functions of PABP.

## Author Summary

The regulation of mRNA translation and stability is of paramount importance for almost every cellular function. In eukaryotes, the poly(A) binding protein (PABP) is a central regulator of both global and mRNA-specific translation. PABP simultaneously interacts with the 3′ poly(A) tail of the mRNA and the eukaryotic translation initiation factor 4G (eIF4G). These interactions circularize the mRNA and stimulate translation. PABP also regulates specific mRNAs by promoting miRNA-dependent deadenylation and translational repression. A key step in understanding PABP's functions is to identify factors that affect its association with the poly(A) tail. Here we show that the cytoplasmic isoform of the mouse heterogeneous nuclear ribonucleoprotein Q (hnRNP-Q2/SYNCRIP), which exhibits binding preference to poly(A), interacts with the poly(A) tail by default when PABP binding is inhibited. In addition, hnRNP-Q2 competes with PABP for binding to the poly(A) tail. Depleting hnRNP-Q2 stimulates translation in cell-free extracts and in cultured cells, in agreement with its function as translational repressor. In addition, hnRNP-Q2 impeded miRNA-mediated deadenylation and repression of target mRNAs, which requires PABP. Thus, competition from hnRNP-Q2 provides a novel mechanism by which multiple functions of PABP are regulated. This regulation could play important roles in various biological processes, such as development, viral infection, and human disease.

## Introduction

Proteins that form dynamic multiprotein complexes with eukaryotic mRNAs play important roles in the control of gene expression [1]. The composition and architecture of messenger ribonucleoprotein particles (mRNPs) largely determines their distribution between different subcellular structures (i.e., polysomes, stress granules, or processing bodies), and ultimately the rates of mRNA translation and degradation [2]. Recent analysis revealed an unexpectedly broad repertoire of mammalian mRNA-binding proteins with largely unknown functions [3]. During translation initiation, which is the rate-limiting and most regulated step of protein synthesis, the 80S ribosome is recruited to the mRNA and positioned at the initiation codon [4]. This process is facilitated by the binding of eukaryotic initiation factor 4E (eIF4E) to the m^7^G cap structure at the 5′ end of the mRNA and poly(A) binding protein (PABP) to the 3′ poly(A) tail. The stimulatory effects of the cap structure and the poly(A) tail on translation are synergistic. eIF4E is a subunit of the eIF4F complex, which also includes eIF4A, an RNA-dependent ATPase/RNA helicase, and eIF4G, a high-molecular-weight scaffolding protein [5]. eIF4G interacts with PABP [6]–[8] and eIF3, which bridges between the eIF4G and 40S ribosomal subunit [9]. These interactions circularize the mRNA [10] and enhance translation (reviewed in [11]–[13]).

PABP is a highly evolutionarily conserved protein, which was first described, to our knowledge, four decades ago [14]. It contains four RNA recognition motifs (RRMs) and a proline-rich C-terminal region, which is involved in protein–protein interactions. PABP binds poly(A) in a cooperative manner and a periodicity of ∼27 nucleotides [15]. The 3′ end-associated PABP is a critical determinant of translational activity of an mRNA, which acts *in cis* [16]. Depletion of PABP from translation extracts decreases the binding of eIF4E to the cap structure and dramatically inhibits 48S and 80S ribosome initiation complex formation [17]. PABP might also play roles at the late step of initiation by promoting ribosomal subunit joining [18], and during termination and ribosome recycling by forming a complex with eRF3 [19]. These results underscore the importance of PABP for global translation. In a different role, PABP enhances the association of the microRNA-induced silencing complex (miRISC) with specific mRNAs to augment miRNA-mediated translation repression [20]. Finally, PABP regulates mRNA deadenylation, which is the first and generally rate-limiting step of mRNA degradation [21]. We and others recently showed that miRISC, which includes the Argonaute (AGO) and GW182 proteins [22]–[24], binds PABP (via GW182) and recruits CNOT7/CAF1 deadenylase to promote poly(A) tail shortening [25],[26]. These and other studies implicate PABP in mRNA-specific regulation of protein synthesis [27]. Intriguingly, PABP is a subject of posttranslational modifications, whose functional significance remains to be established [28].

Two PABP-interacting proteins, Paip1 and Paip2, modulate PABP activity in translation. Paip1 is a positive regulator of translation [29],[30]. In contrast, Paip2 inhibits translation by displacing PABP from the poly(A) tail and eIF4G [31],[32]. The dissociation of PABP from the poly(A) tail would be expected to remodel the mRNP. Here, we report the interaction of mouse heterogeneous nuclear ribonucleoprotein Q isoform 2 (hereafter referred to as hnRNP-Q2) with the poly(A) tail. We also show that hnRNP-Q2 competes with PABP for poly(A) binding to inhibit global protein synthesis both in vitro and in vivo and attenuate miRNA-mediated repression of mRNAs. These findings implicate hnRNP-Q2 in the control of the multifunctional activities of PABP.

## Results

### Paip2-Induced Remodeling of mRNP

**Figure 1:**
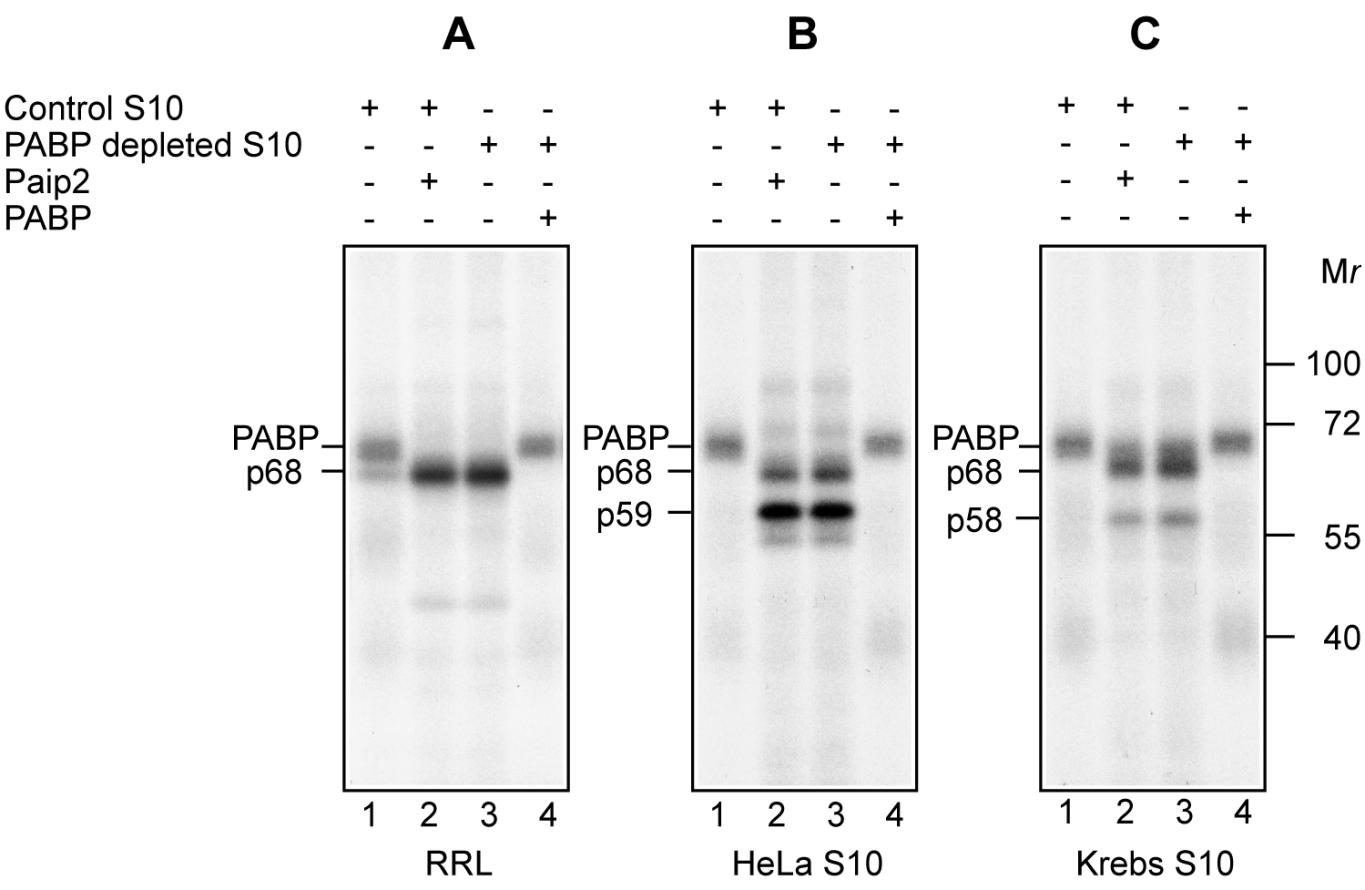
Detection of poly(A) interacting proteins using UV crosslinking. RRL (A), HeLa (B), or Krebs (C) S10 cytoplasmic extracts were subjected to UV-induced crosslinking with the ^32^P-poly(A) tail. The extracts that were either mock-depleted (Control S10) or depleted of PABP were incubated with ^32^P poly(A) tail-labeled globin mRNA at 32°C for 10 min. Prior to adding mRNA, the reaction mixtures were pre-incubated at 32°C for 2 min with GST-Paip2 (20 µg/ml) or PABP (5 µg/ml) as indicated. After UV irradiation and RNase treatment, labeled proteins were analyzed by SDS-PAGE and autoradiography. The positions of molecular mass markers are indicated on the right. Western blotting with an anti-PABP antibody was used to confirm sufficient depletion of PABP from the extracts (for representative analyses, see [54]). Of note, in this and other UV crosslinking analyses, PABP appeared as a fussy band, most probably because RNase digestion does not produce homogenous cross-linked RNA fragments.

Paip2 dramatically decreases the affinity of PABP for poly(A) in model systems containing poly(A) and pure recombinant proteins [31]. To examine the effect of Paip2 on PABP-poly(A) complex formation under more physiological conditions—that is, in the context of cell extracts—we used UV-induced crosslinking, which is a reliable technique to detect specific RNA–protein interactions [33]. For this assay, rabbit globin mRNA was 3′ end extended using [α-^32^P]ATP and yeast poly(A) polymerase. The mRNA was incubated with micrococcal nuclease-treated rabbit reticulocyte lysate (RRL), HeLa, or Krebs cell-free S10 extract, UV irradiated, and digested with a mixture of RNases. Proteins bound to the poly(A) tail were analyzed by SDS-PAGE and autoradiography. In all extracts, almost exclusive crosslinking of a ∼70 kDa polypeptide (p70) to poly(A) was observed (Figure 1A, B, C; lane 1). Adding Paip2 prevented the crosslinking of p70, indicating the dissociation of the p70-poly(A) complex (Figure 1A, B, C; lane 2). These results strongly suggest that p70 is PABP. Indeed, p70 was not detected in extracts that were depleted of PABP using GST-Paip2 affinity matrix (Figure 1A, B, C; lane 3) [34]. Surprisingly, we observed binding of novel proteins (p68 and p58/59) to the poly(A) tail following the sequestration of PABP by Paip2 or PABP depletion (Figure 1A, B, C; lanes 2, 3). Supplementing PABP-depleted extracts with recombinant PABP abolished the binding of these proteins to poly(A) (Figure 1A, B, C; lane 4). Interestingly, p68 appeared as a single prominent band in RRL that was supplemented with Paip2 or depleted of PABP (Figure 1A, lanes 2, 3). p68 was also detectable in control RRL, albeit as a faint band (Figure 1A, lane 1). Consequently, we wished to identify this protein.

### Identification of p68 as hnRNP-Q

To determine the identity of p68 in HeLa cells, a HeLa cytoplasmic extract was depleted of PABP and subjected to chromatography on poly(A)-Sepharose. After washing the beads with buffer containing 0.2 M KCl, poly(A) interacting proteins were sequentially eluted with buffers containing 1 M KCl and 2 M LiCl and resolved by SDS-PAGE (Figure S1A). In contrast to UV crosslinking, this assay captured many proteins, most likely because a great deal of them bind to the sugar-phosphate backbone of the RNA or other RNA-binding proteins. In agreement with the role of ionic RNA–protein interactions, the pattern of poly(A) binding proteins in a more stringent (2 M LiCl) wash was less complex than in 1 M KCl wash. Two candidates for p68 (polypeptides of ∼69 kDa and ∼70 kDa; bands 1 and 2, respectively) were excised from the gel, digested with trypsin, and analyzed by mass spectrometry. One protein in band 1 was identified as hnRNP-Q (UniProtKB accession number O60506), with 36.3% sequence coverage by 22 unique peptides (Table S1). Band 2 was identified as heat shock cognate 71 kDa protein (HSP7C, UniProtKB accession number P11142) with 50.3% sequence coverage by 24 unique peptides (unpublished data). To determine whether any of the two proteins is the poly(A)-crosslinkable p68, we performed immunoprecipitation of p68 from RRL following UV-induced crosslinking. A monoclonal antibody against hnRNP-Q (18E4) efficiently precipitated ^32^P-labeled p68 (Figure S1B). In contrast, two antibodies against hsp70 as well as an antibody against PABP failed to do so. The p68 protein of Krebs extract was also precipitated with the anti-hnRNP-Q antibody (unpublished data). These results demonstrate that p68 is identical to hnRNP-Q.

hnRNP-Q, also termed as NS1-Associated Protein-1 (NSAP1) [35], is an abundant and ubiquitously expressed protein [36] that has been assigned functions in pre-mRNA splicing and mRNA metabolism [37]–[40] as well as a role in IRES-mediated translation [41]–[45]. hnRNP-Q is highly homologous to hnRNP-R and contains an N-terminal acidic domain, three RRMs, and an RGG-rich C-terminal region, which may be involved in RNA binding and protein–protein interactions [37]. Multiple hnRNP-Q isoforms (seven in humans and two in mouse) are derived from alternative splicing of a single gene [37]. Posttranslational modifications of hnRNP-Q, which include phosphorylation and methylation, may determine its subcellular localization and RNA-binding properties [46],[47]. In mouse, the small (562 amino acid long) splicing variant of hnRNP-Q, referred to as SYNaptotagmin-binding Cytoplasmic RNA-Interacting Protein (SYNCRIP) or hnRNP-Q isoform 2 (hnRNP-Q2; accession number NP_062770.1), is mostly cytoplasmic, while the longer hnRNP-Q isoform 1 is in the nucleus [40],[46],[48]. The human ortholog of mouse hnRNP-Q2 is hnRNP-Q isoform 6 (hnRNP-Q6; accession number NP_001153149.1), whose sequence is identical to that of hnRNP-Q2 except for alanine instead of serine in position 357. Notably, the cytoplasmic isoforms of hnRNP-Q contain one, instead of two, nuclear localization signal [37]. We found that RRL contains a single isoform of hnRNP-Q that co-migrates with the smallest isoform of hnRNP-Q of Krebs or HeLa cells (Figure S1C). To determine whether the cytoplasmic hnRNP-Q isoform is associated with actively translated polysomal mRNAs or inactive mRNPs, a HeLa cytoplasmic extract was centrifuged through a sucrose density gradient. Proteins from each fraction were analyzed by Western blotting using antibodies against hnRNP-Q and PABP. Significantly, despite sharing high binding affinity for poly(A), hnRNP-Q and PABP differed with respect to their subcellular distribution. HnRNP-Q was present in the unbound protein/free mRNP fractions (along with eIF4A, eIF4E, and Paip2), as well as in the 40S ribosomal subunit fraction, but not in polysome fractions (Figure S2). In contrast, PABP and the mRNA packaging protein YB-1 [49] associated with polysomes in addition to their presence in the upper gradient fractions. These results indicate the association of hnRNP-Q with untranslated mRNPs, and are consistent with other studies on the subcellular distribution of NSAP1/hnRNP-Q, PABP, and YB-1 [42],[45],[50],[51].

### Competition Between hnRNP-Q2 and PABP for Poly(A) Binding

**Figure 2:**
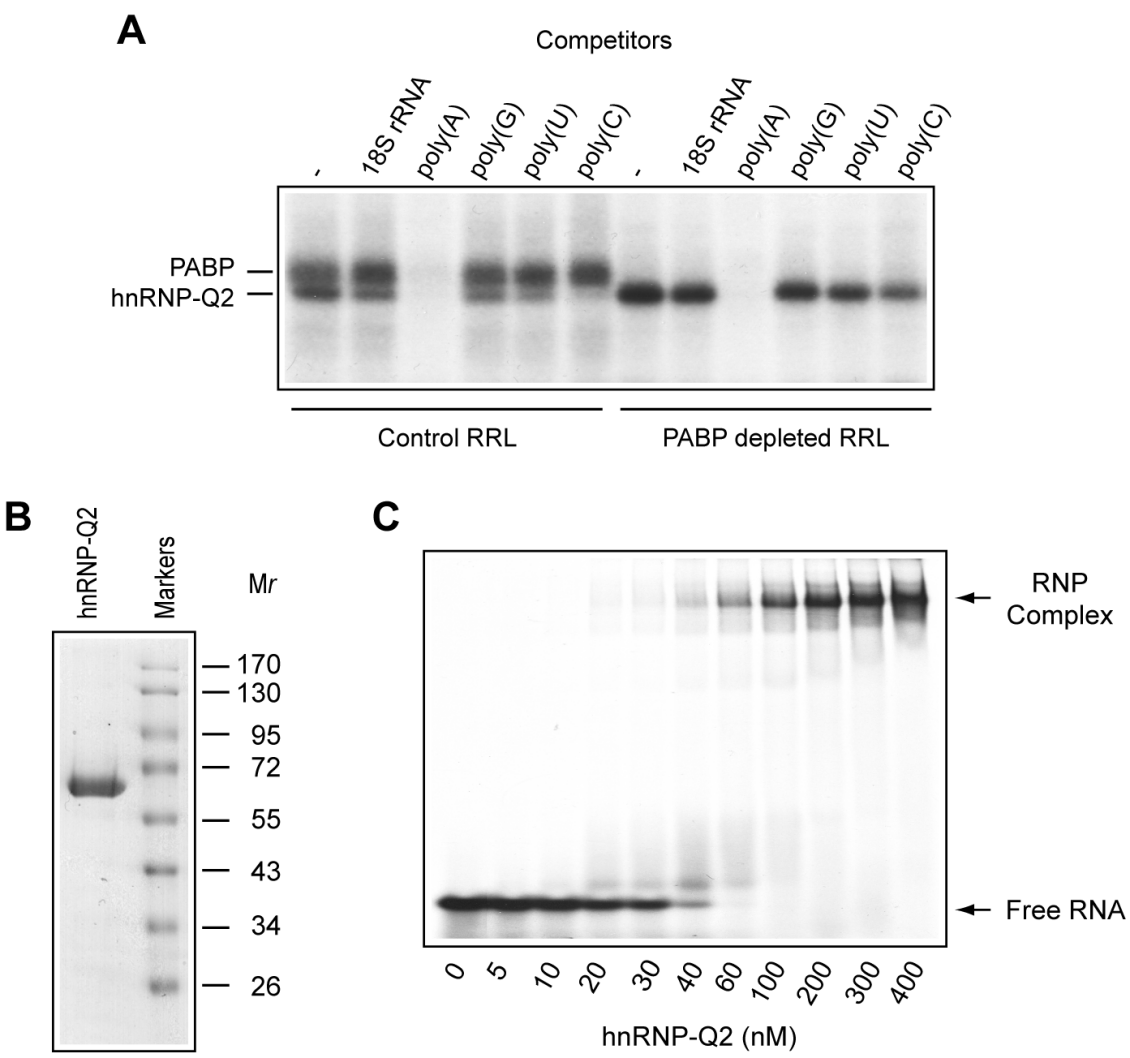
Specificity of binding of hnRNP-Q2 to the poly(A) tail. (A) Proteins of control or PABP-depleted RRL UV crosslinked with the ^32^P-poly(A) tail were analyzed by SDS-PAGE as described for Figure 1. RNA competitors, 18S rRNA, poly(A), poly(G), poly(U), and poly(C), were included in the reaction mixtures at 5 µg/ml concentrations. (B) Recombinant hnRNP-Q2 (2 µg) was analyzed by SDS-PAGE and Coomassie blue staining. Prestained molecular mass markers (MBI Fermentas) are shown in the right lane. (C) High-affinity binding of hnRNP-Q2 to oligo(A). EMSA was performed as described in Materials and Methods. For each lane, a constant small amount of ^32^P-oligo(A)_30_ RNA was incubated with the indicated concentrations of hnRNP-Q2. The *K*d value of ∼30 nM was calculated from three independent experiments.

Interestingly, it has been reported that SYNCRIP/hnRNP-Q2 exhibits preferential binding to poly(A) [48]. Consequently, we wished to investigate the poly(A) binding specificity of hnRNP-Q2 by performing RNA competition experiments in RRL. The addition of poly(A) (5 µg/ml) to a PABP-depleted RRL completely inhibited UV crosslinking of hnRNP-Q2 to the poly(A) tail (Figure 2A). Poly(G), poly(U), and 18S ribosomal RNA (rRNA) had no effect on the crosslinking of hnRNP-Q2, while poly(C) was slightly inhibitory. Poly(A) also specifically inhibited crosslinking of PABP to the poly(A) tail, as assayed in control (mock-depleted) RRL. Thus within RRL, hnRNP-Q2 exhibits preference for poly(A).

To quantitatively characterize the hnRNP-Q2/poly(A) interaction, a bacterially expressed His-tagged mouse hnRNP-Q2 was affinity purified by Ni^2+^-NTA agarose chromatography. SDS-PAGE and UV spectrum analyses revealed that the hnRNP-Q2 preparation is largely free of contaminating proteins and nucleic acids (Figure 2B and unpublished data). The recombinant hnRNP-Q2 was used in an electrophoretic mobility shift assay (EMSA), in which a constant small amount of 5′ ^32^P-labeled oligo(A_30_) was titrated with increasing amounts of hnRNP-Q2. Following incubation, RNA/protein complexes were separated from free oligo(A_30_) by native gel electrophoresis and quantified using a phosphorimager (Figure 2C). The apparent *K*d for hnRNP-Q2 (which equals to the protein concentration at which 50% of the probe is shifted into a complex) was estimated to be ∼30 nM. Interestingly, the amount of the shifted probe gradually increased with the increased amounts of hnRNP-Q2, indicating that stable complex formation requires cooperative binding of hnRNP-Q2. Thus, hnRNP-Q2 binds avidly to oligo(A), albeit less strongly than PABP, for which a *K*d of 4–7 nM has been reported [52],[53].

**Figure 3:**
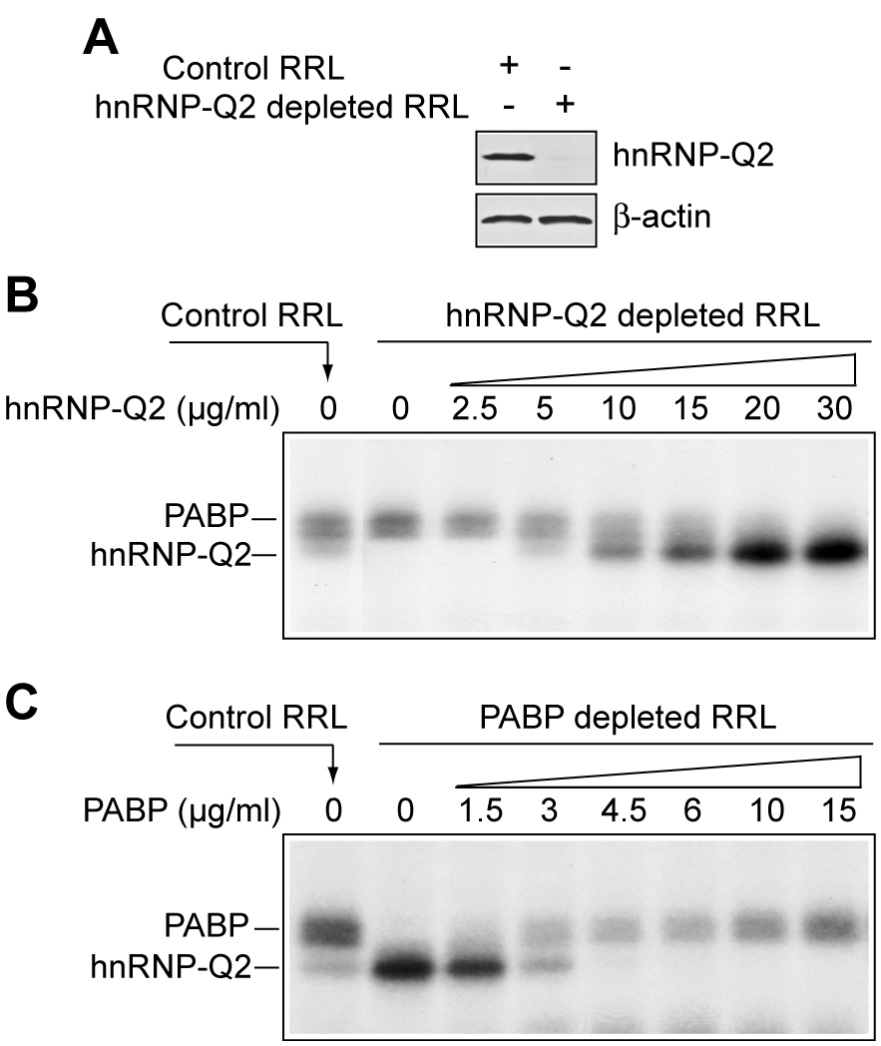
Competition between hnRNP-Q2 and PABP for binding to the poly(A) tail in RRL. (A) Western blot analysis of RRL depleted with either anti-FLAG (Control RRL) or anti-hnRNP-Q antibody. The blot was probed for anti-hnRNP-Q or anti-β-actin (loading control). (B) Proteins of control or hnRNP-Q2-depleted RRL that crosslink with the ^32^P-poly(A) tail in the presence of the indicated concentrations of recombinant hnRNP-Q2. (C) Proteins of control or PABP-depleted RRL that crosslink to the ^32^P-poly(A) tail in the presence of the indicated concentrations of recombinant PABP.

To determine whether hnRNP-Q2 competes with PABP for poly(A) binding, it was depleted from RRL using the anti-hnRNP-Q antibody. Western blotting revealed efficient (∼90%) depletion of hnRNP-Q2 but not β-actin, which served as a loading control (Figure 3A). In the absence of endogenous hnRNP-Q2, efficient crosslinking of PABP to the globin mRNA poly(A) tail occurred (Figure 3B). Adding increasing amounts of hnRNP-Q2 gradually diminished the crosslinking of PABP. In a reciprocal experiment, PABP-depleted RRL [54] was supplemented with increasing concentrations of recombinant PABP. In the absence of PABP, hnRNP-Q2 was bound by the poly(A) tail by default, appearing as a prominent band (Figure 3C). The hnRNP-Q2 band gradually faded away, while the PABP band intensified, when increasing concentrations of PABP were added to the reaction mixture. These results clearly demonstrate that PABP and hnRNP-Q2 compete with each other for poly(A) binding.

### HnRNP-Q2 Is an Inhibitor of PABP/Poly(A)-Dependent Translation

**Figure 4:**
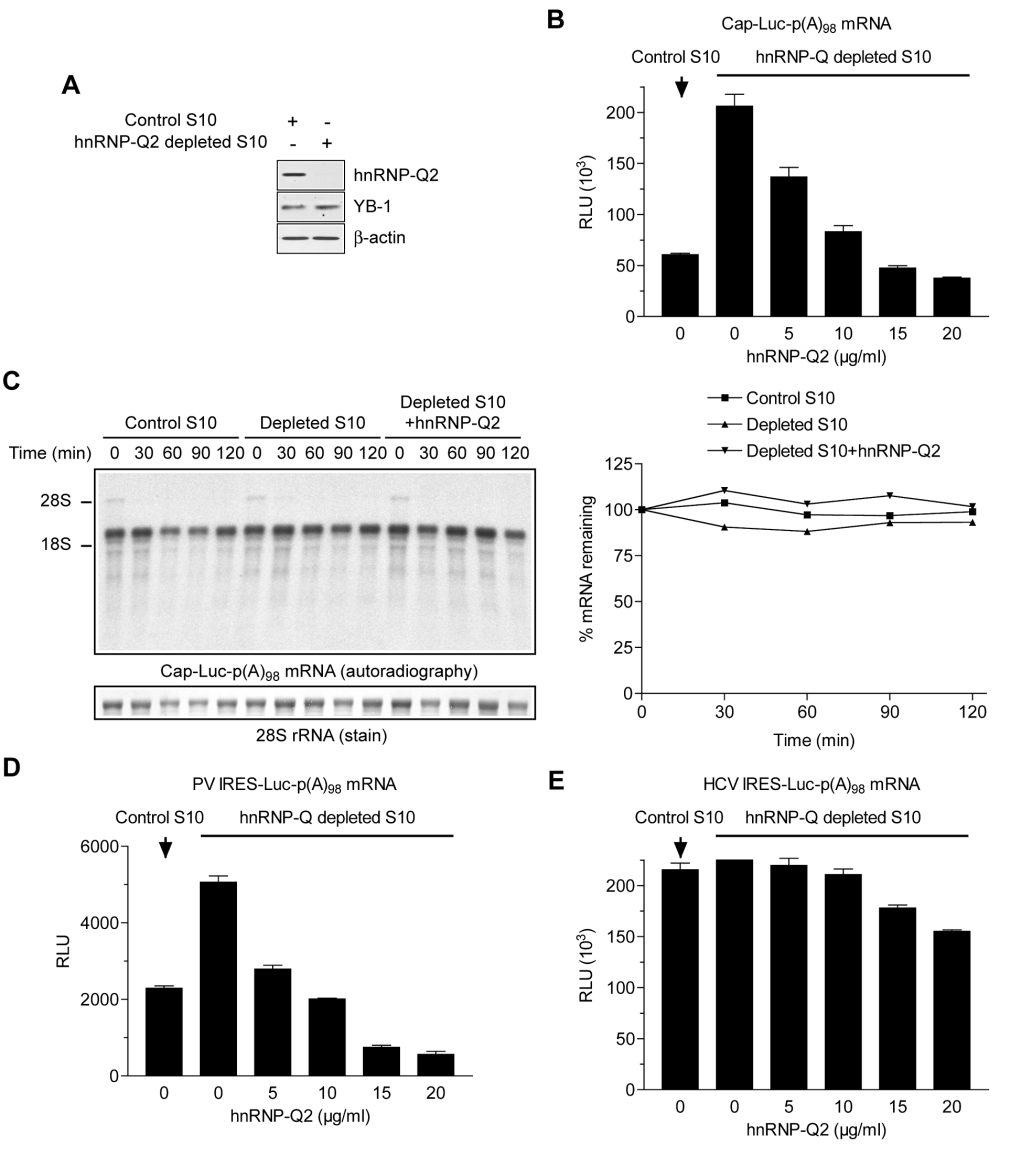
HnRNP-Q2-mediated inhibition of translation in Krebs extracts. (A) Western blot analysis of Krebs S10 extracts depleted with either anti-FLAG (Control S10) or anti-hnRNP-Q antibody. The blot was probed for hnRNP-Q, YB-1, or β-actin (loading control). (B) Mock-depleted (control) or hnRNP-Q2-depleted S10 Krebs extracts were programmed with Cap-Luc-p(A)_98_ mRNA in the absence or presence of the indicated concentrations of hnRNP-Q2. (C) Stability of Cap-Luc-p(A)_98_ mRNA. ^32^P-labeled Cap-Luc-p(A)_98_ was used to program mock-depleted (control) or hnRNP-Q2-depleted S10 Krebs extracts not supplemented or supplemented with recombinant hnRNP-Q2 (30 µg/ml), as indicated. Total RNA was isolated at the indicated times from the aliquots of the reaction mixture, resolved by formaldehyde-agarose gel electrophoresis, and transferred to a membrane. Cap-Luc-p(A)_98_ mRNA and 28S rRNA were detected by autoradiography (left panel, top) and staining (left panel, bottom), respectively. Cap-Luc-p(A)_98_ mRNA band intensities were determined and corrected for loading by 28S rRNA (right panel). The values for Cap-Luc-p(A)_98_ mRNA recovered at the beginning of incubation were set as 100%. (D and E) Control (mock depleted) and hnRNP-Q2-depleted Krebs extracts were programmed with PV IRES-Luc-p(A)_98_ (D) or HCV IRES-Luc-p(A)_98_ (E) mRNAs. hnRNP-Q2 was added to the reaction mixtures at the indicated concentrations. On panels B, D, and E, the data are averages of three assays with standard deviations from the mean.

Having shown that hnRNP-Q2 and PABP compete for binding to the poly(A) tail, we predicted that hnRNP-Q2 would counteract PABP activity in translation. To investigate this, endogenous hnRNP-Q2 was immunodepleted from Krebs extract (∼90% depletion; Figure 4A). Depletion of hnRNP-Q2 stimulated the translation of capped and polyadenylated (A_98_) luciferase mRNA [designated as Cap-Luc-p(A)_98_ mRNA], by ∼3.5-fold (Figure 4B). The stimulatory effect of hnRNP-Q2 depletion on translation was not due to co-depletion of YB-1 (Figure 4A), an mRNA packaging protein and a general repressor of translation [49],[54],[55]. Adding back hnRNP-Q2 to the depleted extract decreased translation, and this inhibition was hnRNP-Q2 dose-dependent. It is noteworthy that the amounts of hnRNP-Q2 added in this and other assays were in the range of the concentrations normally found in Krebs extracts (∼30 µg/ml; Figure S3). To rule out the possibility that hnRNP-Q2 inhibits protein synthesis by destabilizing mRNA, the decay of ^32^P-labeled Cap-Luc-p(A)_98_ mRNA was monitored in translation extracts either containing or lacking hnRNP-Q2. Cap-Luc-p(A)_98_ mRNA remained intact in control and hnRNP-Q2-depleted translation extracts over a 2 h incubation period (Figure 4C). Furthermore, adding hnRNP-Q2 (30 µg/ml) to the depleted extract had no effect on the stability of Cap-Luc-p(A)_98_ mRNA.

We next examined the effect of hnRNP-Q2 on cap-independent translation driven by different viral internal ribosome binding sites (IRESs). The hnRNP-Q2-depleted extract was ∼2.2-fold more active than mock-depleted extract in supporting translation from the poliovirus (PV) IRES (Figure 4D). However, the translation from the hepatitis C virus (HCV) IRES (which is PABP and eIF4G-independent, in contrast to PV IRES) was not significantly augmented by hnRNP-Q depletion (Figure 4E). Consistent with these results, in hnRNP-Q2-depleted extract, PV IRES exhibited greater susceptibility to inhibition by recombinant hnRNP-Q2, as compared to HCV IRES (Figure 4, compare panels D and E). Thus, competition from hnRNP-Q2 does not significantly affect the function of ribosomes and translational factors other than the PABP/eIF4G complex.

### HnRNP-Q Inhibits eIF4F Recruitment to the mRNA

**Figure 5:**
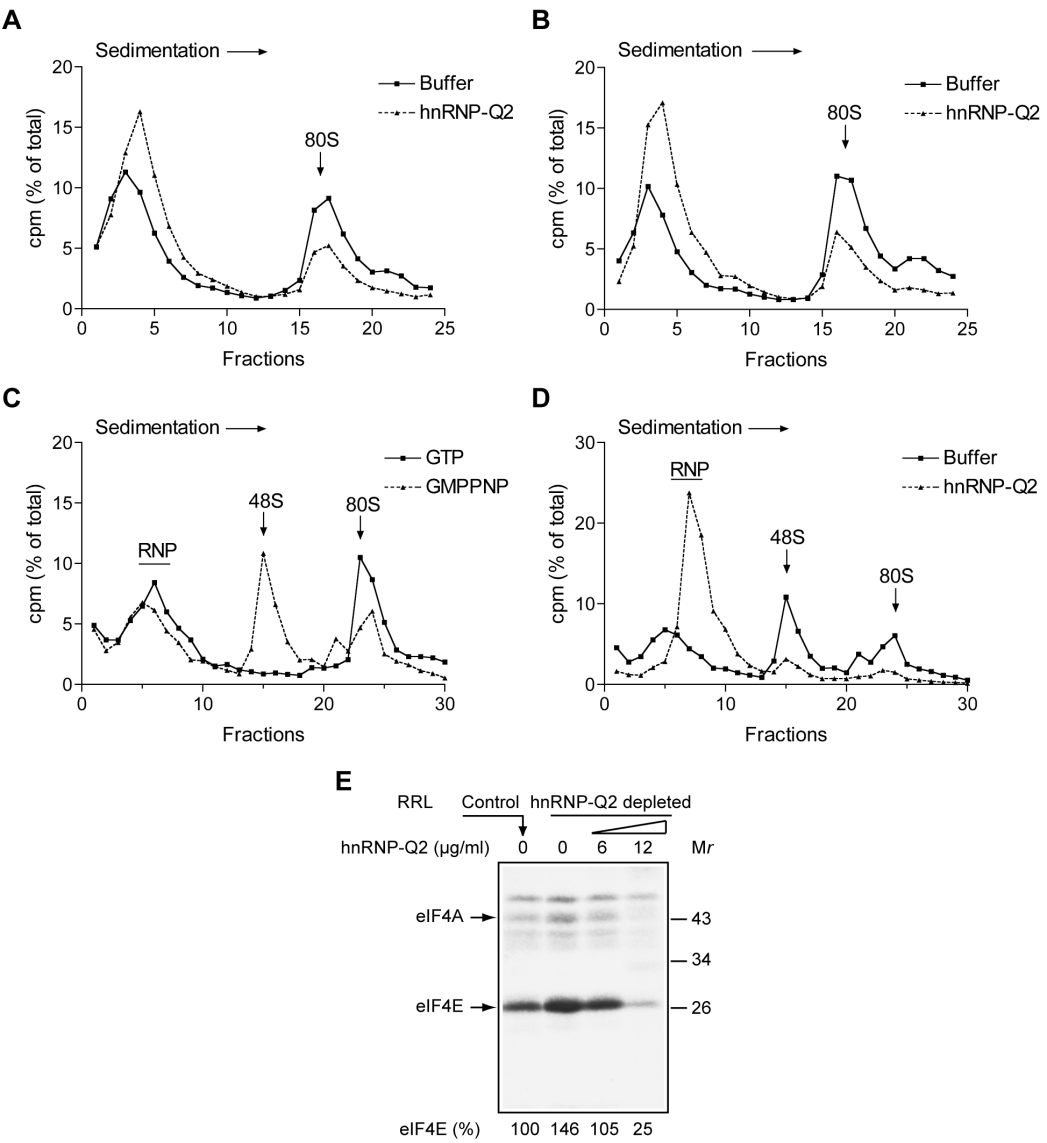
HnRNP-Q2 inhibits m^7^G cap structure recognition by translation initiation factors. (A–D) Inhibition of 80S and 48S initiation complex formation by hnRNP-Q2 in nuclease-treated RRL. 80S ribosome binding to 3′ end labeled globin mRNA was assayed in a cycloheximide (0.6 mM)-supplemented RRL, normal (A) or hnRNP-Q2-depleted (B), in the presence of control buffer (squares) or recombinant hnRNP-Q2 (15 µg/ml) (triangles). (C) Validation of the 48S pre-initiation complex formation in the presence of GMPPNP. GTP or GMPPNP were added to the reaction mixtures at 2 mM final concentration as indicated. Other conditions were similar to those described for panel B. (D) 48S pre-initiation complex formation in hnRNP-Q2-depleted RRL in the presence of GMPPNP and either control buffer (squares) or hnRNP-Q2 (25 µg/ml) (triangles). The reaction mixtures were analyzed on 5-ml 15%–30% (A and B) or 11-ml 10%–30% (C and D) sucrose gradients. (E) HnRNP-Q2 dose-dependent inhibition of eIF4E binding to the m^7^G cap structure in RRL as analyzed by chemical crosslinking. Control and hnRNP-Q2-depleted RRL were incubated with oxidized ^32^P-cap-labeled poly(A)-extended Luc mRNA in the absence or presence of the indicated concentrations of recombinant hnRNP-Q2. The positions of eIF4E and eIF4A are indicated. Relative efficiencies of eIF4E crosslinking are indicated at the bottom (the value obtained for control RRL was set as 100%).

To elucidate whether competition from hnRNP-Q2 targets the initiation step of translation, we examined ribosome binding using commercial nuclease-treated RRL. Although cap- and poly(A) tail dependence of RRL is decreased after nuclease treatment [56],[57], a significant dependence on these structures for translation is observed at low levels of input mRNA and elevated potassium ion concentrations (Figure S4) [54],[58]–[60]. For instance, at 60 mM additional KCl concentration, capping and polyadenylation enhance the translation of Luc mRNA (0.5 µg/ml) by 12.5- and 3.3-fold, respectively (Figure S4B, D). Therefore, the assays below were carried out using KCl (60 mM)-supplemented RRL and low (<0.5 µg/ml) mRNA concentrations. To investigate the formation of 80S ribosome initiation complex, RRL was incubated with radiolabeled globin mRNA in the presence of cycloheximide. The 80S complex was resolved from the unbound mRNA by sucrose gradient centrifugation. The addition of hnRNP-Q2 (20 µg/ml) to control or hnRNP-Q2-depleted RRL inhibited 80S initiation complex formation by 2.3-3-fold (Figures 5A, B). A similar reduction of 80S ribosome recruitment in the presence of hnRNP-Q2 was observed in normal or hnRNP-Q2-depleted Krebs extracts (Figure S5). To determine whether hnRNP-Q2 also targets 48S pre-initiation complex formation, 60S ribosomal subunit joining was inhibited by GMPPNP, a nonhydrolysable GTP analog [17]. In GMPPNP supplemented RRL, the labeled mRNA redistributed from the 80S fractions of the gradient to the 48S fractions, thereby validating the assay (Figure 5C). Importantly, adding hnRNP-Q2 (24 µg/ml) to hnRNP-Q2-depleted RRL inhibited 48S initiation complex formation by ∼5-fold with a profound shift of mRNA to the RNP fractions (Figure 5D). To determine whether hnRNP-Q2 inhibits translation prior to 48S complex formation, we examined the interaction of eIF4E with the cap-structure in mock- and hnRNP-Q2-depleted RRL by chemically crosslinking the lysates with polyadenylated Luc mRNA ^32^P-labeled at the 5′ cap-structure. We earlier showed that this assay provides a highly reliable measure of eIF4F activity [54]. In hnRNP-Q2-depleted RRL, eIF4E crosslinking was enhanced ∼1.5-fold relative to mock-depleted RRL (Figure 5E). Adding increasing concentrations of hnRNP-Q2 decreased crosslinking in a dose-dependent manner (to 25% of control). Thus, hnRNP-Q2 impairs the interaction of eIF4E with the cap-structure. Since PABP stimulates eIF4E-cap interaction [17],[61], it is most probable that hnRNP-Q2 acts by inhibiting this function of PABP. To gain evidence that hnRNP-Q2 targets eIF4-group factors in Krebs extract, purified eIF4F, eIF4A, eIF4E, and eIF4B were added to this system either lacking or containing hnRNP-Q2. These factors stimulated the translation of Cap-Luc-p(A)_98_ mRNA, consistent with their presence in limiting amounts in Krebs extracts (Figure S6 and [62]). In agreement with the partial repression of eIF4F activity by hnRNP-Q2, exogenous eIF4F relieved hnRNP-Q2-mediated translation inhibition (from 5.5- to 1.4-fold). eIF4A, eIF4E, and eIF4B also relieved the inhibition of translation by hnRNP-Q2, albeit less efficiently than eIF4F.

### Long Poly(A) Tails Augment hnRNP-Q2-Mediated Translational Repression

**Figure 6:**
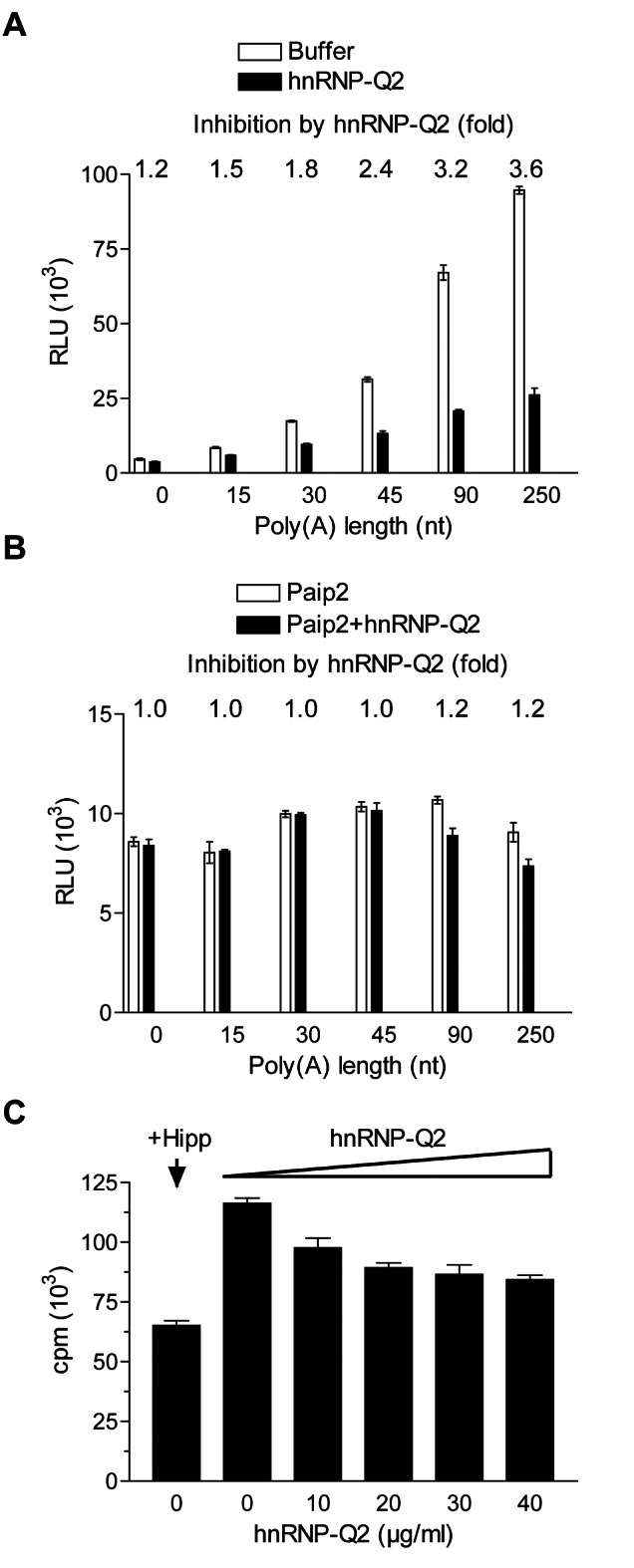
Poly(A) tail length and PABP-dependent inhibition of translation by hnRNP-Q2 in Krebs extract. (A) Krebs extract that was not nuclease-treated was programmed with Cap-Luc mRNAs (0.2 µg/ml) bearing poly(A) tails of the indicated length. Control buffer of hnRNP-Q2 (20 µg/ml) was added to the reaction mixtures as indicated. (B) Sequestering of PABP by Paip2 renders translation insensitive to the poly(A) tail length and inhibition by hnRNP-Q2. Cap-Luc mRNA with increasing poly(A) tails was translated in the untreated extract in the presence of Paip2 (15 µg/ml) as described for panel A. hnRNP-Q2 (20 µg/ml) was added to the reaction mixtures where indicated. Inhibition of translation by hnRNP-Q2 is shown on the top of the panels. (C) Endogenous [^35^S]methionine incorporation in the untreated extract in the presence of the indicated concentrations of hnRNP-Q2 or 10 µM hippuristanol (Hipp). Incubation was at 32°C for 2 h. Average values for trichloroacetic acid-insoluble radioactivity in 1-µl aliquots of the samples from three assays with standard deviations are shown.

The length of the poly(A) tail determines the number of PABP molecules bound to an mRNA, thereby indirectly controlling PABP-dependent translation. To study whether hnRNP-Q2-mediated inhibition of translation is dependent on the length of the poly(A) tail, we compared the effect of hnRNP-Q2 on the translation of Luc mRNAs either without (A_0_) or with a poly(A) tail of increasing length (A_15_, A_30_, A_45_, A_90_, and A_250_). For these studies we used Krebs extract that was not nuclease-treated. In several studies, the omission of nuclease treatment during the preparation of extracts has been proven ideal for mimicking cap-poly(A) synergy and other translational control mechanisms operating in vivo [56],[63]–[65]. As shown in Figure 6A, the untreated extract was strikingly poly(A) tail dependent, exhibiting up to 20-fold stimulation of translation by poly(A) tailing. Importantly, adding hnRNP-Q2 to the extract inhibited the translation of mRNAs with long (90–250 nt) poly(A) tails more strongly (3.2–3.6-fold) than the translation of the mRNA with short (15–30 nt) poly(A) tails (1.5–1.8-fold), while having a marginal effect on the translation of the poly(A-) mRNA (1.2-fold inhibition). To assure that the displacement of PABP from the poly(A) tail is required for the hnRNP-Q2-mediated translational inhibition, PABP was sequestered into the PABP–Paip2 complex. In the presence of Paip2, low-efficient PABP-independent translation was virtually insensitive to inhibition by hnRNP-Q2 (Figure 6B). Inactivation of PABP also abolished the response of translation to poly(A) length. Thus, the translational inhibition by hnRNP-Q2 in the nuclease untreated extract is both PABP and poly(A) tail dependent. To determine to what extent endogenous hnRNP-Q2 inhibits the translation of polyadenylated mRNAs, we attempted to deplete the untreated extract of hnRNP-Q2. However, we failed to achieve substantial immunodepletion of hnRNP-Q2 using the 18E4 antibody (unpublished data). It is possible that hnRNP-Q2 cannot interact with this antibody when bound to mRNA that is in untreated extract. Since the assays above employed the extract that was not nuclease treated, it was of interest to test the effect of hnRNP-Q2 on the translation of endogenous mRNAs (Figure 6C). HnRNP-Q2 reduced ^35^S-methionine incorporation in the untreated extract in a dose-dependent manner. However, this inhibition was relatively small (up to 1.4-fold), as compared to that of the exogenous mRNA translation (Figure 6A). It is likely that re-initiation of translation in the untreated extract is less efficient than in intact cells; hence, ^35^S-methionine incorporation primarily reflects the rate of polypeptide chain elongation on the preformed polysomes. Consistent with this notion, inhibiting re-initiation of translation with hippuristanol [66] only modestly (1.8-fold) reduced ^35^S-methionine incorporation (Figure 6C).

### HnRNP-Q2 Knockdown Stimulates Protein Synthesis in Vivo

**Figure 7:**
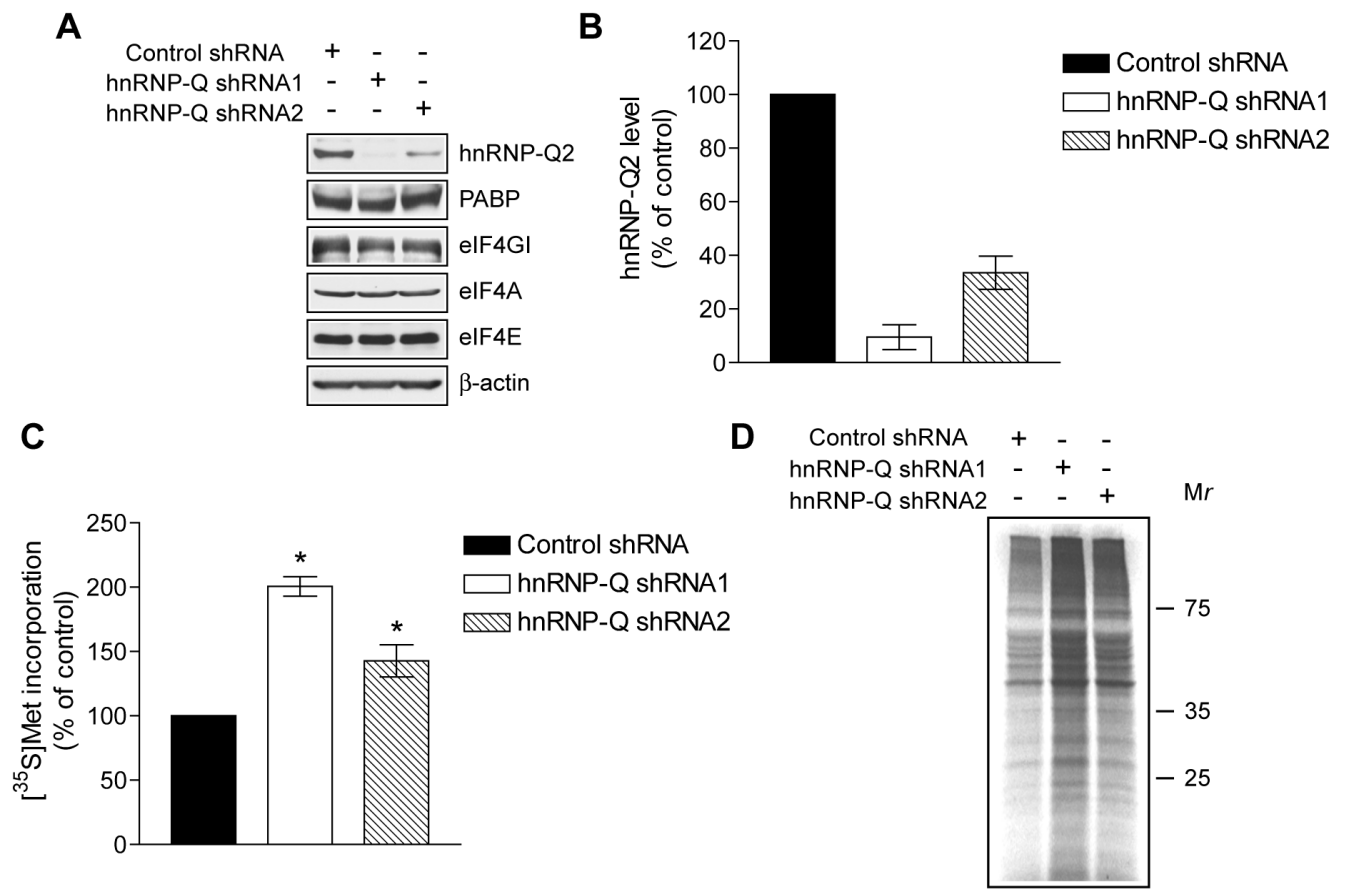
HnRNP-Q2 silencing stimulates global protein synthesis. L929 cells were infected with lentiviruses expressing the nontarget control shRNA or shRNAs (1 and 2) against hnRNP-Q. (A) Cytoplasmic extracts of control and hnRNP-Q knockdown cells equalized for protein content were subjected to Western blotting for hnRNP-Q2, PABP, eIF4GI, eIF4A, eIF4E, and β-actin, as indicated. (B) Quantitative analysis of hnRNP-Q2 bands in panel A using NIH ImageJ software. The values were normalized by those of β-actin. The value in control was set as 100%. The data are means with standard deviations from three experiments. (C) Protein synthesis in control and hnRNP-Q2-knockdown L929 cells as analyzed by [^35^S]methionine/cysteine labeling. The mean values for ^35^S incorporation into proteins from three independent assays with standard deviations are shown as percentages of the value in control (* *p*<0.025). (D) Representative patterns of ^35^S-labeled proteins from panel C. Proteins were resolved by SDS 12% PAGE and detected by autoradiography.

Next, we investigated whether hnRNP-Q2 inhibits protein synthesis in vivo by reducing the amount of hnRNP-Q2 in the mouse fibroblast-like cell line L929 using shRNA. One shRNA against hnRNP-Q (shRNA1) caused significant silencing of hnRNP-Q2 (∼90%; Figure 7A, B). Another shRNA (shRNA2) was less effective in hnRNP-Q2 silencing (∼75%). No changes in the levels of PABP, eIF4GI, eIF4A, and eIF4E were found. Overall translation rate was measured by [^35^S]methionine/cysteine incorporation into newly synthesized proteins. shRNA1 expressing cells showed a ∼2-fold increase in incorporation as compared to cells expressing nontargeting control shRNA (Figure 7C). hnRNP-Q knockdown by shRNA2 evoked less potent stimulation of translation, as compared to shRNA1 (∼1.4-fold). SDS-PAGE analysis of newly synthesized proteins indicated that hnRNP-Q2 inhibits global protein synthesis (Figure 7D).

### HnRNP-Q2 Antagonizes PABP Activity in miRNA-Mediated Deadenylation

**Figure 8:**
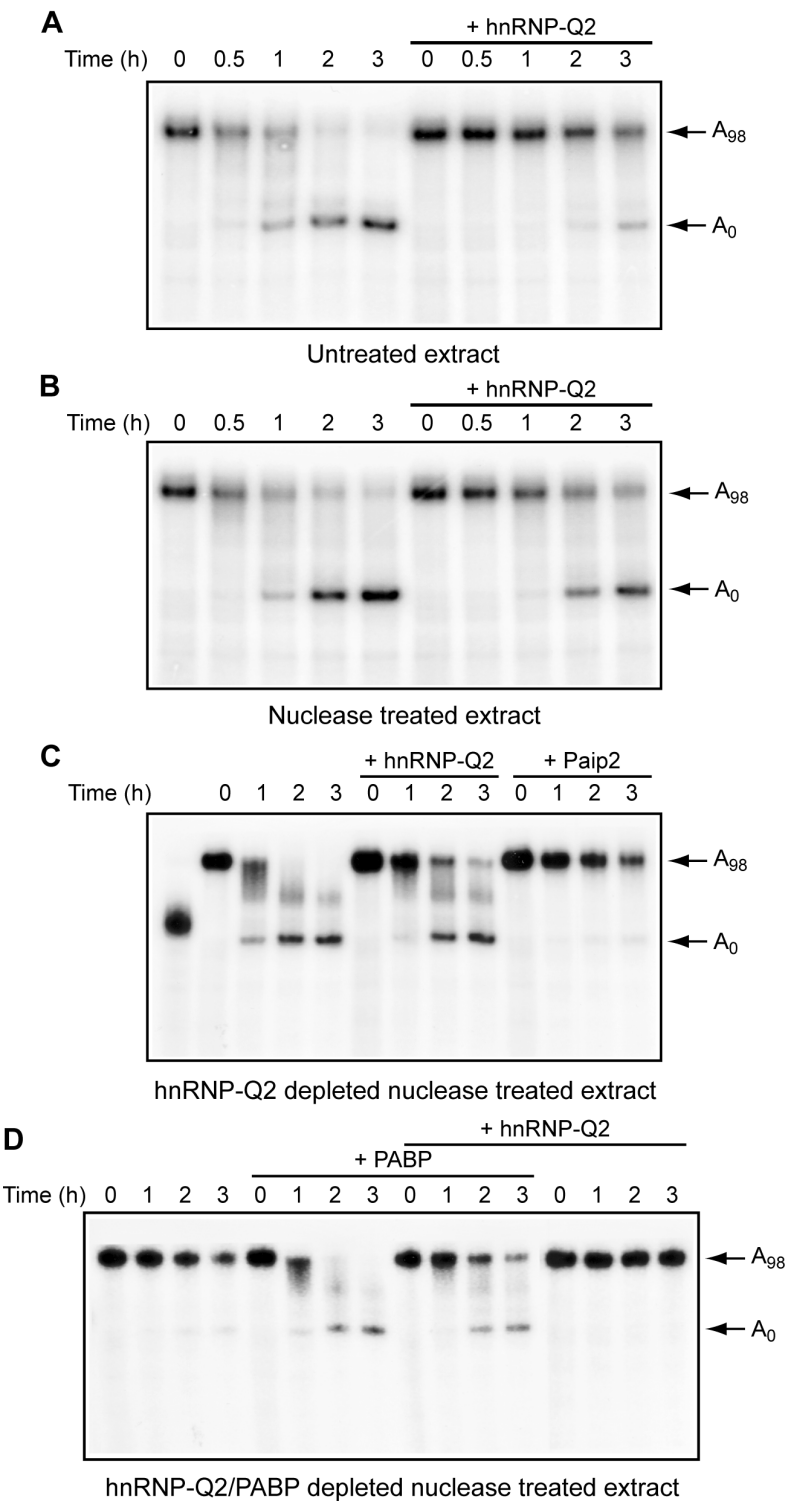
HnRNP-Q2 counteracts PABP function in let-7a miRNA dependent deadenylation. Kinetics of deadenylation of 6xB-3′UTR RNA in untreated (A) or nuclease-treated (B) S10 Krebs extracts. Recombinant hnRNP-Q2 (50 µg/ml) was added to the reaction mixtures where indicated. (C) Kinetics of deadenylation of 6xB-3′UTR RNA in hnRNP-Q2-depleted Krebs extract. Recombinant hnRNP-Q2 (36 µg/ml) or GST-Paip2 (16 µg/ml) were included in the reaction mixtures where indicated. 6xB-3′UTR RNA treated with RNase H in the presence of oligo(dT) is shown in lane 1. (D) Kinetics of deadenylation of 6xB-3′UTR RNA in hnRNP-Q2 and PABP double-depleted Krebs extract. Recombinant PABP (6 µg/ml) and hnRNP-Q2 (36 µg/ml) were included in the reaction mixtures either alone or in combination as indicated. The positions of intact and deadenylated RNAs are indicated on the right. The data are the representative of three independent experiments.

MiRNAs, in addition to inhibiting translation, mediate deadenylation and decay of target mRNAs [67]. PABP facilitates miRNA-dependent deadenylation through its interaction with the GW182-CAF1/CCR4 deadenylase complex [25],[68]. We wished to determine whether hnRNP-Q2 antagonizes this function of PABP in a Krebs extract, which faithfully recapitulates PABP-dependent miRNA-mediated deadenylation [25]. An RNA bearing six let-7a targets sites and a 98 nucleotide long poly(A) sequence (6xB-3′UTR RNA), labeled uniformly with ^32^P UTP [25], was extensively deadenylated by Krebs extracts (Figures S7 and 8). The completely deadenylated (A_0_) RNA is likely unstable as it appeared as a less prominent band as compared to input (A_98_) RNA. The formation of the A_0_ RNA species was dependent on let-7a miRNA as it was blocked by the addition of anti-let-7a 2′-*O*-methylated oligonucleotide (2′-*O*-Me) and also not observed with a reporter bearing mutations in nucleotides complementary to the let-7a “seed” sequence (6xBMut-3′UTR RNA) (Figure S7). To investigate how deadenylation is affected by competition between PABP and hnRNP-Q2, hnRNP-Q2 was added to untreated or nuclease-treated Krebs extracts and the kinetics of deadenylation of 6xB-3′UTR RNA was followed. As expected, exogenous hnRNP-Q2 inhibited the conversion of the full-length A_98_ RNA into A_0_ RNA (Figure 8A, B). The effect of hnRNP-Q2 on deadenylation was somewhat stronger in the untreated extract, most likely because a fraction of PABP is withdrawn from competition as being sequestered by endogenous mRNAs. To better assess the effects of PABP and hnRNP-Q2 on deadenylation, we made use of extracts that were depleted of these proteins. In hnRNP-Q2-depleted extract, the full-length A_98_ RNA disappeared and the A_0_ RNA was formed within a 2-h incubation period (Figure 8C). Consistent with the importance of PABP for poly(A) tail shortening [25], the addition of Paip2 almost abrogated deadenylation. Importantly, adding hnRNP-Q2 markedly impaired deadenylation, as ∼30% of RNA retained the full-length poly(A) tail after 2 h of incubation. This demonstrates that competition from hnRNP-Q2 inhibits miRNA-mediated deadenylation. To determine whether hnRNP-Q2 also interferes with the function of exogenous PABP in miRNA-mediated deadenylation, the assay was carried out in Krebs extract devoid of both hnRNP-Q2 and PABP. In this extract, a vast proportion of the input RNA retained the poly(A) tail during the time course of reaction (Figure 8D). However, almost all the RNA became deadenylated within 2 h after addition of recombinant PABP. Importantly, adding back hnRNP-Q2 to the PABP-supplemented extract markedly decreased the rate of deadenylation. Under these conditions a significant fraction of RNA (∼25%) remained intact even after 3 h of incubation. In addition, the feeble deadenylation of the RNA in PABP and hnRNP-Q2 double-depleted extract (which could be due to incomplete depletion of PABP) was prevented by the addition of recombinant hnRNP-Q2. Taken together, these results demonstrate that hnRNP-Q2 stabilizes mRNAs by antagonizing PABP activity in miRNA-mediated deadenylation.

### HnRNP-Q2 Depletion in Cultured Cells Augments miRNA-Mediated Repression

**Figure 9:**
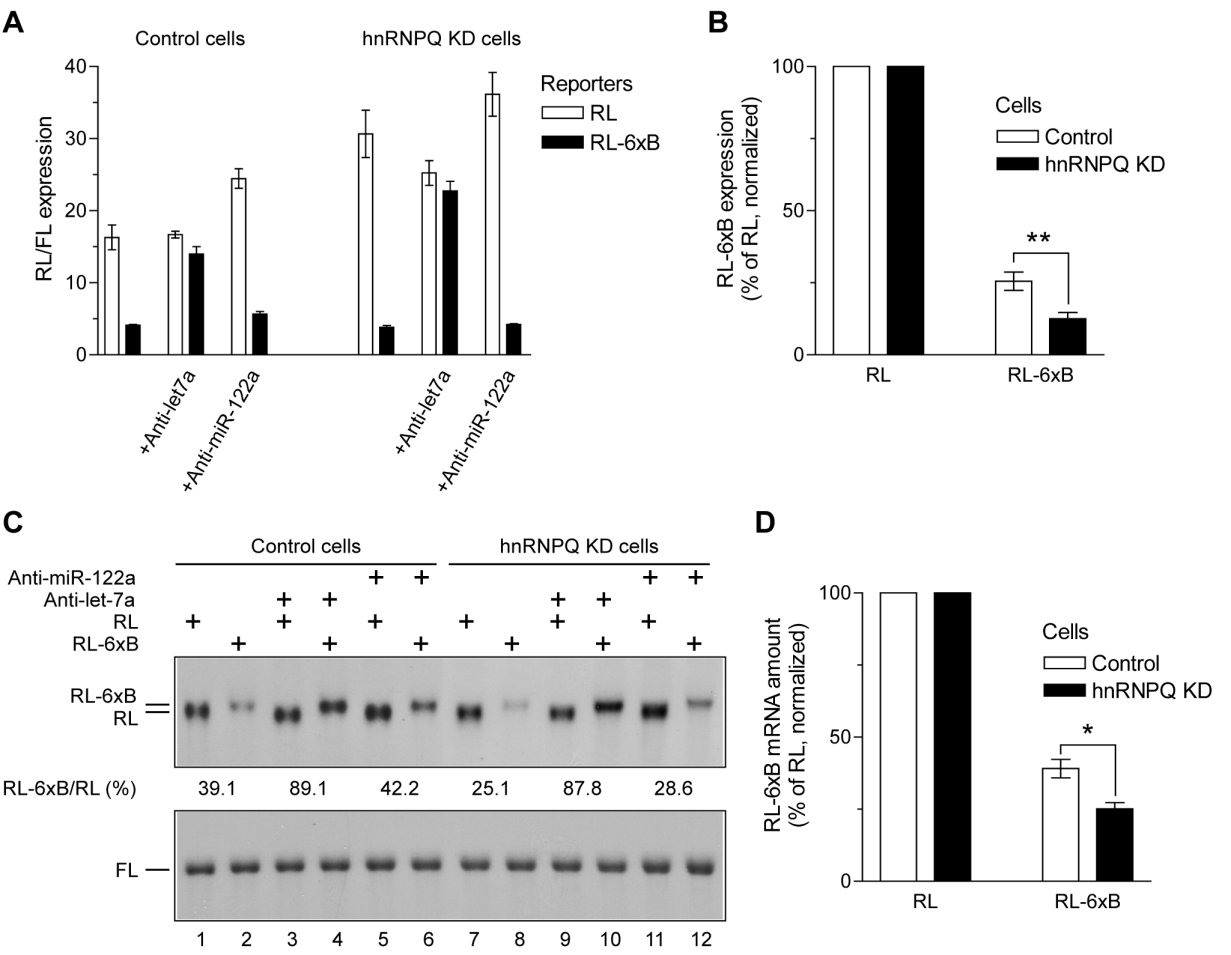
HnRNP-Q2 depletion augments miRNA-mediated repression in cultured cells. (A) Activities of RL and RL-6xB reporters in control and hnRNP-Q2 knockdown L929 cells. Cells expressing control shRNA or hnRNP-Q shRNA2 were transfected with RL reporters either possessing or lacking the 6xB sequence in parallel with a Firefly luciferase (FL) reporter. Anti-let-7a or anti-miR-122a (negative control) 2′-*O*-Me oligonucleotides were cotransfected where indicated. Two days after transfection, activities of RL and FL were measured and their ratio was determined. (B) The expression of RL-6xB relative to RL (which was set as 100% in both control and hnRNP-Q2-knockdown cells). (C) Equal amounts (10 µg) of RNA from transfected cells were analyzed by Northern blotting using probes specific for RL (top) and FL (bottom) reporters. The bands were quantified using a Typhoon PhosphorImager (GE Healthcare). To correct for loading and transfection efficiency, the values for RL and RL-6xB mRNAs were normalized to those for FL mRNA. The RL-6xB/RL ratio for each condition is given under the top panel. Note that RL-6xB mRNA migrates slightly slower than RL mRNA due to the presence of the 6xB sequence. (D) The levels of RL-6xB mRNA in control and hnRNP-Q KD cells were normalized to those of RL mRNA (which were set as 100% for both conditions). In (A), (B), and (D) the values are means from three transfections with standard deviations (* *p*<0.025, ** *p*<0.001).

Next, we examined whether hnRNP-Q2 reduces miRNA-induced repression in vivo. Control and hnRNP-Q2 knockdown L929 cells were transfected with *Renilla* luciferase (RL) reporters, with or without six let-7a miRNA target sites (6xB) [69]. A firefly reporter (FL) was co-transfected to normalize for transfection efficiency. In control cells, the expression of RL-6xB was ∼4-fold lower than RL (Figure 9A, B). Importantly, hnRNP-Q2 knockdown significantly augmented this inhibition (from 4-fold to 8.1-fold). Co-transfection of anti-let-7a 2′-*O*-Me oligonucleotide, but not control anti-miR-122a oligonucleotide, dramatically reduced the inhibition of RL-6xB expression, consistent with the role of let-7a miRNA in silencing of the RL-6xB reporter (Figure 9A). We determined the amount of RL-6xB mRNA to be ∼2.6-fold and ∼4-fold lower than RL mRNA in control and hnRNP-Q knockdown cells, respectively (Figure 9C, top; compare lane 2 with 1 and lane 8 with 7; Figure 9D). This difference in the relative RL-6xB levels can partially explain the augmented reduction of expression of RL-6xB reporter after hnRNP-Q2 depletion (Figure 9B). Attesting to the dependence of RL-6xB mRNA decay on let-7a miRNA, the levels of RL-6xB mRNA were rescued by co-transfection of anti-let-7a, but not anti-miR-122a, 2′-*O*-Me oligonucleotide (Figure 9C).

## Discussion

In eukaryotic cells, the association of PABP with the poly(A) tail stimulates global translation [12],[17], but also promotes miRNA-dependent deadenylation and repression of target mRNAs [20],[25],[70]. Paip2 inhibits these functions of PABP by dissociating the PABP-poly(A) complex [31]. In this study, we applied UV-induced crosslinking to characterize the composition of the poly(A) mRNP in the absence of PABP. Upon UV irradiation, proteins crosslink to poly(A) when bound in proximity to the photochemically reactive purine rings [33],[71]. In contrast, ionic interactions of proteins with the sugar-phosphate backbone of poly(A) do not fulfill the requirement for crosslinking. In addition, UV irradiation does not cause protein–protein crosslinking. Thus, UV-induced crosslinking is a reliable technique in revealing specific protein–poly(A) interactions. In all cell extracts studied, PABP appeared as the single major poly(A)-binding protein and Paip2 decreased the association of PABP with poly(A) (Figure 1 and [31],[71]). When PABP was depleted from RRL, hnRNP-Q2 became the major poly(A) binding protein by default. PABP and hnRNP-Q2 are presumably the only strong poly(A) binders since RRL depleted of both PABP and hnRNP-Q2 produced no major cross-links (unpublished data). To our knowledge, the first description of a cytoplasmic poly(A) interacting protein (p78X) that is distinct from PABP dates back to 1981 [72]. At the time, the identity of this protein and its function has not been explored. Along with hnRNP-Q2/Q6, p58/59 crosslinked with poly(A) in PABP-depleted Krebs and HeLa extracts (Figure 1B, C). This protein(s) might be similar or identical to the nuclear poly(A)-associated protein p60A with as yet unidentified function [72]. The absence of p58/59 from rabbit reticulocytes, which lack nuclei, favors this possibility. The leakage of p58/59 from the nucleus might have occurred during extract preparation, as these proteins are especially abundant in extracts derived from excessively disrupted cells (unpublished data).

How important is hnRNP-Q2 for mRNA translation and metabolism? Preferential binding to poly(A) distinguishes hnRNP-Q2 from the bulk of general RNA-binding proteins, which do not exhibit sequence specificity [73]. Moreover, hnRNP-Q2 competed with PABP for binding to the poly(A) tail of the mRNA. This competition would be expected to impair multiple functions of PABP in global and mRNA-specific regulation of protein synthesis. In agreement with this prediction, we showed that hnRNP-Q2 inhibits the initiation of translation that requires the PABP/eIF4G complex. In addition, hnRNP-Q inhibited miRNA-mediated deadenylation and repression of mRNAs that are promoted by PABP.

A paramount issue in addressing the competition between hnRNP-Q2 and PABP for poly(A) binding in vivo concerns the relative abundance of these proteins in the cell. The concentration of hnRNP-Q2 in Krebs and RRL translation mixtures is ∼1.7-fold higher than that reported for PABP (240–480 nM versus 140–280 nM, Figure S3, and [25],[54]). Since the affinity of hnRNP-Q2 for poly(A) is ∼6-fold lower than that of PABP, its molar excess over PABP might not be sufficient for efficient competition under standard physiological conditions. However, a significant fraction of PABP might be sequestered into complexes with repressor proteins, such as Paip2. This would not only increase the hnRNP-Q2/PABP ratio but also impair PABP cooperative binding that is important for the stability of the PABP/poly(A) complex. Finally, the cytosolic hnRNP-Q levels are likely elevated in the G2/M phases of cell cycle and under stress conditions [44],[74]. As a result of these rearrangements, conditions for efficient competition from hnRNP-Q could be met. The observed stimulatory effects of hnRNP-Q2 depletion on translation both in vitro and in vivo indicate that the endogenous concentration of hnRNP-Q2 suffices for translational inhibition. Although SDS-PAGE analysis of proteins de novo indicates that hnRNP-Q2 targets global protein synthesis (Figure 7D), it might also differentially affect the translation of specific mRNAs. Pointing to this possibility is the negative regulation of RhoA mRNA translation by the cytoplasmic isoform of hnRNP-Q [36], and the presence of NSAP1/hnRNP-Q in a translational silencing complex that recognizes a specific element in the 3′UTR of inflammatory mRNAs (termed IFN-γ-Activated Inhibitor of Translation, or GAIT, element) [75],[76]. On the other hand, binding of hnRNP-Q to several IRES elements stimulates translation [41]–[45]. In addition, hnRNP-Q can possibly activate IRESs indirectly by reducing competition from the bulk of cellular mRNA. Finally, as shown here and discussed below, the displacement of PABP from the poly(A) tail by hnRNP-Q2 attenuates miRNA-induced deadenylation, decay, and repression of specific mRNAs. Thus, in addition to its function as a general translation repressor, hnRNP-Q might play divergent roles in mRNA-selective translational control.

Translationally repressed mRNAs accumulate in two cytoplasmic foci: processing bodies and stress granules, which serve as sites for mRNA degradation or storage [77],[78]. It is conceivable that once bound by hnRNP-Q2, the mRNA is guided to cytoplasmic granules. Indeed, in neurons, SYNCRIP/hnRNP-Q2 localizes to mRNA granules that are transported along dendrites [79]. In addition, in stressed cells, hnRNP-Q relocalizes to cytoplasmic granules, as evidenced by its co-localization with HSP70, GW182, and TIA-1 marker proteins [74]. In both types of granules mRNA translation is inhibited [2]. It is an intriguing possibility that hnRNP-Q2 plays a role in this inhibition.

Deadenylation and subsequent decrease of target mRNA levels significantly contributes to miRNA-induced reduction of gene expression [67],[70]. PABP interacts with the GW182 proteins, which are essential components of the miRISC [25]. This interaction promotes miRNA-dependent deadenylation, potentially by bringing the poly(A) tail in proximity to miRISC-associated CAF1/CCR4 deadenylase. HnRNP-Q2 markedly impaired PABP-dependent let-7a miRNA-mediated deadenylation in Krebs extract, most probably by partially displacing PABP from the poly(A) complex (Figure 8). In a more physiological context, L929 cells, hnRNP-Q2 depletion augmented the miRNA-dependent degradation and repression of a target mRNA (Figure 9). Interestingly, the expression of RL-6xB reporter was stronger affected by hnRNP-Q2 depletion at the level of protein than mRNA. Thus, it is likely that hnRNP-Q2 also targets the function of PABP in miRNA-mediated translational repression [20]. It is noteworthy that the role of hnRNP-Q2/NSAP1/SYNCRIP in mRNA stabilization is also suggested by its presence in protein complexes that stabilize c-*fos* and c-*myc* proto-oncogene mRNAs [38],[80]. Thus, competition from hnRNP-Q provides a novel mechanism by which multiple functions of PABP are regulated. Control of PABP functions by hnRNP-Q2 could play important roles in various biological processes, such as development, virus infection, and human disease.

## Materials and Methods

### Proteins and Antibodies

Recombinant PABP, GST-Paip2, eIF4A, eIF4E, and eIF4B were expressed and purified as described [17],[31],[58],[81]. Native eIF4F was purified from RRL [81]. The proteins were dialyzed against buffer A containing 20 mM Tris-HCl, pH 7.5, 100 mM KCl, 0.1 mM EDTA, 1 mM DTT, and 10% glycerol. Mouse monoclonal anti-hnRNP-Q (clone 18E4) antibody and anti-FLAG antibody used for the preparation of hnRNP-Q2-depleted and mock-depleted Krebs extracts, respectively, were from Sigma. For the description of antibodies used for Western blotting and immunoprecipitation, see corresponding sections below.

### Cloning, Expression, and Purification of hnRNP-Q2

The expression vector for mouse hnRNP-Q2 was constructed as follows. The cDNA encoding hnRNP-Q2 (accession number GI:114145481) was obtained by reverse transcription-PCR (RT-PCR) of Krebs-2 cell poly(A)^+^ RNA using QIAGEN OneStep RT-PCR kit. The hnRNP-Q2 coding DNA fragment was amplified with forward (GATATACCATGGCTACAGAACATGTTAATGGAAATGGTACTGAAGAGCCTATGGATACTACTTCAGCAG) and reverse (GTGGTGCTCGAGTTGTAACAGGTCAGGACCGGCCTCG) primers, designed to generate the 5′ terminal *Nco*I and 3′ terminal *Xho*I restriction sites and eliminate an internal *Nco*I site by introducing a silent mutation (the flanking sequences for cloning purposes and silent mutation are underlined). After digestion with *Nco*I and *Xho*I, the hnRNP-Q2 coding DNA fragment was cloned into *Nco*I-*Xho*I sites of pET28a (Novagen) to generate a vector for the expression of hnRNP-Q2 with a six-His sequence at the C-terminus (pET28a-hnRNP-Q2-His). The His-tagged hnRNP-Q2 protein was expressed in *Escherichia coli* and purified to apparent homogeneity by Ni^2+^-nitrilotriacetic acid agarose chromatography (QIAGEN) using a batch procedure. Briefly, frozen bacterial cells were suspended in suspension buffer (S) (20 mM HEPES, pH 7.5, 5 mM 2-mercaptoethanol, and 10% glycerol) containing 2 M KCl and Complete EDTA-free protease inhibitor cocktail (Roche) and lysed by sonication. Following addition of Triton X-100 (to 0.1% final concentration), cell debris was removed by centrifugation (40,000 *g*, 1 h, and 4°C). The supernatant was supplemented with 20 mM imidazole, pH 7.5 and applied to Ni^2+^-NTA agarose resin equilibrated with buffer S containing 20 mM imidazole, 2 M KCl, and 0.1% Triton X-100. The beads were washed first with the same buffer and then with buffer S containing 20 mM imidazole, 0.1 M KCl, and 0.1% Triton X-100. Bound proteins were eluted with buffer S containing 250 mM imidazole and 0.1 M KCl and dialyzed against buffer A.

### mRNA Preparation

Plasmids for transcription of Luc mRNAs with 98-nucleotide long poly(A) tails, T3luc(A)^+^, T7PVluc(A)^+^, and T7HCVluc(A)^+^ [82], were linearized with *Bam*HI and transcribed with T3 or T7 RNA polymerase, as appropriate. The templates for transcription of Luc mRNAs with variable poly(A) tails were obtained by PCR using plasmid T3luc [82], forward primer GCTCGAAATTAACCCTCACTAAAGGG, and five different reverse primers, collectively named as (T)_n_GGATCCCCCGGGCTGC, where (T)_n_ designates oligo(dT) tracts of 0, 15, 30, 45, and 90 nucleotides (the core T3 promoter sequence is underlined). After purification on a Chroma Spin-1000 column (BD Biosciences), the PCR products were transcribed with T3 RNA polymerase. Luc mRNA with the poly(A) tail of ∼250 nucleotides was obtained by polyadenylation of the Luc-A_98_ mRNA using yeast poly(A) polymerase (USB) as recommended by the manufacturer. Capping of mRNAs was done using the ScriptCap m^7^G capping system (Epicentre). The integrity of mRNAs was verified by denaturing agarose gel electrophoresis.

### EMSA

Synthetic RNA oligonucleotide (A_30_, Dharmacon) was 5′ labeled using [γ-^32^P]ATP and polynucleotide kinase. Prior to use, the probe was purified by centrifugation through a Chroma Spin-10 column. Standard binding reaction mixtures (20 µl) contained 8 fmol (∼40,000 cpm) of 5′-labeled A_30_, 10 µl of 2× incubation buffer (40 mM HEPES-KOH, pH 7.3, 200 mM KCl, 4 mM MgCl_2_, 2 mM DTT, 0.1% NP40, 10% glycerol, and 0.2 mg/ml acetylated bovine serum albumin), and 2 µl of hnRNP-Q2 diluted to the appropriate concentrations with buffer A. Following incubation at 30°C for 30 min, the samples were supplemented with 2 µl of 50% v/v glycerol and analyzed by electrophoresis in 7% nondenaturing polyacrylamide gel (prepared with TBE buffer and 5% v/v glycerol) at 4°C. Bands were visualized by autoradiography. The amount of free and bound RNA was determined using a Typhoon Phosphorimager (GE Healthcare).

### In Vitro Translation

Krebs extracts, untreated or treated with micrococcal nuclease, were prepared as described previously [34]. Where indicated, the nuclease-treated extracts were depleted or mock depleted of hnRNP-Q2 and PABP as described below. The reaction mixtures (12 µl) included Krebs extracts (50% by volume), salts, essential translation components [34], indicated mRNAs (0.2 µg/ml, unless specified in the figure legends), and proteins. For optimal translation of HCV and PV IRES-containing mRNAs, the concentration of KOAc in the reaction mixtures was increased by 75 mM. Incubation was at 32°C for 1 h. Luciferase activity was determined in 1 µl aliquots of samples using Luciferase assay system (Promega) and Lumat LB 9507 bioluminometer (EG&G Bertold). The relative light units (RLU) reported are averages of three assays with the standard deviation from the mean.

### Depletion of Krebs Extract and RRL of hnRNP-Q2 and PABP

Prior to hnRNP-Q2 depletion, a nuclease-treated Krebs extract was supplemented with salts, amino acids, and energy generating system as described previously [83]. The supplemented Krebs extract or RRL (Promega) were clarified by centrifugation at 10,000 *g* for 1 min. To couple the antibody against hnRNP-Q to beads, 40 µg (∼20 µl) of anti-hnRNP-Q (18E4) were incubated with a Protein-G Sepharose slurry (GE Healthcare; 150 µl pelleted beads per 0.6 ml of phosphate buffered saline [PBS; 137 mM NaCl, 2.7 mM KCl, 10 mM Na_2_HPO_4_, and 2 mM KH_2_PO_4_]) at 4°C for 1.5 h while mixing on a rotator. In a control tube, the beads were similarly incubated with 40 µg of an anti-FLAG antibody. Following incubation, the beads were washed by centrifugation, once with PBS containing 1% bovine serum albumin and twice with buffer D (25 mM HEPES-KOH, pH 7.3, 50 mM KCl, 75 mM KOAc, and 2 mM MgCl_2_) [34]. After the final centrifugation at 2,400 *g* for 2 min, the bead pellets were suspended in 600 µl of Krebs extract (or RRL), already containing the necessary translation components. After gentle agitation for 1.5 h, the beads were precipitated by centrifugation as above. The supernatants, which constitute the hnRNP-Q2 and mock-depleted extracts, were collected, centrifuged again to remove any residual beads, and frozen at −80°C in small aliquots. For the depletion of PABP, the supplemented Krebs extract or RRL was incubated with the GST-Paip2 protein that was immobilized onto glutathione-Sepharose beads [34]. Mock-depleted extracts were treated with GST alone. For the removal of both hnRNP-Q2 and PABP, the extracts were incubated first with anti-hnRNP-Q and then with GST-Paip2. The efficiency of hnRNP-Q2 and PABP depletion was analyzed by Western blotting.

### UV Crosslinking

The poly(A) tail of rabbit globin mRNA (1.5 µg; Gibco BRL, discontinued) was extended in a 50 µl total reaction volume using [α-^32^P]ATP (60 µCi, 3,000 Ci/mmol, Perkin Elmer) and yeast poly(A) polymerase (1,500 U, USB) as recommended by the manufacturer. Incubation was at 37°C for 30 min. After extraction with a mixture of phenol and chloroform, the RNA was purified by Chroma Spin-100 column chromatography. Reaction mixtures (15 µl) contained 10 µl of RRL (or Krebs extract supplemented with essential translational components), labeled RNA (∼400,000 cpm), and other components as indicated in the figure legends. After incubation at 30°C for 10 min, the samples were applied drop-wise along a line onto a Parafilm-covered glass plate and irradiated with UV light at 254 nm (using a 15-W germicidal lamp with ∼4 cm distance between the lamp and the samples) for 20 min on ice. The samples were collected into tubes already containing 4 µl of an RNase cocktail (1 mg/ml RNase A and 1,000 U/ml [∼0.7 mg/ml] nuclease S7 [Roche]). One µl of 200 mM CaCl_2_ was then added, and the samples were digested at 37°C for 30 min. Proteins were denatured by adding 40 µl of 1.5× SDS sample buffer and heating at 95°C for 5 min. Samples were analyzed by SDS-PAGE (10% acrylamide, 99∶1 acrylamide/N, N′-methylenebisacrylamide ratio) and autoradiography at −80°C with an intensifying screen.

### Western Blotting

Primary antibodies were the following: mouse monoclonal anti-hnRNP-Q antibody (clone 18E4, Sigma), rabbit polyclonal anti-PABP antibody [31], rabbit polyclonal anti-Paip2 antibody (Sigma), rabbit polyclonal anti-YB-1 (Abcam), rabbit polyclonal anti-eIF4GI antibody [84], mouse monoclonal anti-eIF4A antibody [85], mouse monoclonal anti-eIF4E antibody (BD Biosciences), mouse monoclonal anti-ribosomal protein S6 antibody (Santa Cruz), and mouse monoclonal anti-β-actin antibody (Sigma). Proteins in the samples were resolved by SDS-PAGE, transferred to a nitrocellulose membrane, and detected using Western Lightning chemiluminescence kit (Perkin-Elmer Life Sciences). Primary antibodies against hnRNP-Q, PABP, eIF4GI, YB-1, and β-actin were used diluted 1∶2,500, 1∶1,000, 1∶1,000, 1∶1,000, and 1∶5,000, respectively. The dilutions of antibodies against Paip2, eIF4E, and ribosomal protein S6 were as per the manufacturers' instructions. Secondary HRP-conjugated anti-mouse or anti-rabbit antibody (GE Healthcare), as appropriate, was used diluted 1∶5,000. Typically, a single membrane was probed, exposed, and stripped before probing with another antibody.

### Immunoprecipitation of p68

Anti-hnRNP-Q and anti-PABP antibodies that were used for Western blotting were also used for immunoprecipitation of p68. Mouse monoclonal and rabbit polyclonal anti-hsp70 antibodies were from Santa Cruz and Calbiochem, respectively. To generate ^32^P-labeled p68, PABP-depleted RRL was UV crosslinked with the ^32^P poly(A) tail-labeled globin mRNA in twenty 15-µl aliquots. The aliquots were combined and treated with RNAses. After the addition of 10% SDS to 0.2% final concentrations, the crosslinked RRL was 10-fold diluted with PBS containing 0.2% NP40. For immunoprecipitation, 0.7 ml portions of the diluted reaction mixture were incubated with antibody-conjugated protein-G Sepharose beads (20 µl) at 4°C for 4 h while mixing on a rotator. The beads were washed three times with 0.2% NP-40 containing PBS and finally with PBS alone. Bound proteins were eluted by heating in SDS-sample buffer and analyzed by SDS-PAGE and autoradiography. UV crosslinked control and PABP-depleted RRL were loaded on the same gel for comparison.

### Subcellular Distribution of hnRNP-Q2

HeLa S3 cells were grown in a 15-cm Petri dish to ∼90% confluence. A polysome profile was obtained after centrifugation of a fresh cellular extract through a 10%–50% sucrose density gradient according to standard methods [86]. Centrifugation was in a Beckman SW41Ti rotor at 35,000 rpm for 2.5 h at 4°C. Optical density at 254 nM was continuously recorded using an ISCO fractionator (Teledyne ISCO, Lincoln, NE). Aliquots of fractions (30 µl) were analyzed by Western blotting using antibodies against hnRNP-Q, PABP, Paip2, YB-1, eIF4A, eIF4E, and 40S ribosomal protein S6.

### Ribosome Binding Assays

For 80S ribosome binding studies, ^32^P-poly(A)-labeled globin mRNA (∼300,000 cpm, 6 ng) was incubated in a total reaction volume of 30 µl with nuclease-treated Krebs extract or KCl (60 mM)-supplemented RRL in the presence of the translation components and 0.6 mM cycloheximide [17]. Where indicated, recombinant hnRNP-Q2 or control buffer were added to the reaction mixtures. Incubation was at 32°C for 15 min. Reactions were stopped by 4-fold dilution with ice-cold HSB buffer [54], and 80S ribosomal complexes were resolved by centrifugation in 5-ml 15%–30% sucrose gradients (Beckman SW55 rotor, 54,000 rpm for 1 h, 45 min at 4°C). Fractions (0.2 ml) were collected from the top of the gradients and analyzed by scintillation counting. 48S complexes were formed in RRL in the presence of GMPPNP (2 mM), MgCl_2_ (2 mM), and cycloheximide (0.6 mM) [17]. Prior to the addition of mRNA, the reaction mixtures were pre-incubated at 32°C for 2 min. Subsequent incubation with ^32^P-poly(A)-labeled globin mRNA was at 32°C for 10 min. Reactions were stopped by chilling and diluting 4-fold with buffer K (20 mM Tris-HCl, pH 8.0, 2 mM DTT, 100 mM potassium acetate) containing 5 mM MgCl_2_ [81]. Total reaction mixtures were applied onto 11-ml 10%–30% sucrose gradients prepared with the same buffer. Centrifugation was in SW41 rotor at 40,000 rpm and 4°C for 3.5 h. Fractions (0.36 ml) were collected from the top of the gradients. Radioactivity in each fraction was determined and expressed as percentage of total recovered counts. The area under the peaks (less background) was used to quantify ribosome binding. Sedimentation profiles of the purified 40S and 60S ribosomal subunits in sucrose density gradients served to confirm the positions of the 80S and 48S initiation complexes.

### Chemical Crosslinking Assay

Uncapped Luc mRNA (Promega) was 3′ poly(A) extended and radioactively labeled at the m^7^G cap using [α-^32^P]GTP, S-adenosyl methionine, and vaccinia-virus guanylyltransferase [54]. After purification and oxidation with NaIO_4_, the ^32^P cap-labeled RNA was used for crosslinking studies in RRL as described previously [17],[54],[87]–[89].

### mRNA Stability Assay

To analyze mRNA stability in vitro, Cap-Luc-A_98_ mRNA, uniformly labeled with [α-^32^P]UTP during transcription (1.6×10^6^ cpm, 40×10^3^ cpm/ng), was translated in Krebs extracts (mock-depleted or hnRNP-Q2-depleted) in a total volume of 100 µl under standard conditions. Fifteen µl aliquots of the reaction mixture were withdrawn at 30-min intervals. Total RNA was deproteinized by phenol-chloroform extraction, separated on a formaldehyde-1% agarose gel, and transferred onto a nylon membrane (Hybond-N; GE Healthcare). Blots were stained with Blot Stain Blue (Sigma) to determine the levels of 28S rRNA (loading control). ^32^P-labeled Cap-Luc-A_98_ mRNA was detected by autoradiography. Quantifications of band intensities were carried out using NIH ImageJ software.

### Mass Spectrometry Analysis

The Coomassie-stained protein bands were cut from the gel and treated with trypsin. Tryptic peptides were analyzed at Genome Quebec Innovation Centre using a nano-HPLC system coupled to a 4000 Q TRAP mass spectrometer (Applied Biosystems, Foster City, CA). Peptide identities were determined by searching UniProt database (version 13.8) with restriction to *human* using Mascot (version 2.1, Matrix Science, London).

### HnRNP-Q2 Knockdown

Mouse fibroblast-like cell line L929 was purchased from ATCC. The cells were transduced with two shRNAs directed against human hnRNP-Q, shRNA1 (TRCN0000112054), shRNA2 (TRCN0000112053), and a nontargeting control shRNA (SHC002) using a lentivirus transduction system (Sigma-Aldrich) as recommended by the manufacturer. Cells were selected with puromycin (2 µg/ml) for 4 d. One week after infection, the cytoplasmic extracts were prepared and analyzed for down-regulation of hnRNP-Q2 by Western blotting.

### Protein Synthesis Analysis

Control and hnRNP-Q2 knockdown L929 cells, at ∼90% confluence, were washed with methionine-free DMEM and incubated in methionine-free DMEM supplemented with dialyzed fetal bovine serum (10%; GIBCO), glutamine, and [^35^S]methionine/cysteine labeling mixture (100 µCI/ml) at 37°C for 30 min. Cells were lysed in SDS-sample buffer, and ^35^S incorporation into trichloroacetic acid-insoluble material was determined [34]. The values for ^35^S incorporation were normalized to the amounts of total protein in the samples.

### Deadenylation Assay

To produce the reporter RNA, a 350 nt DNA fragment of pRL-6xB-A_98_ containing six target sites for human miRNA let-7a [62] was PCR-amplified using primers T7-3′UTR (encoding the T7 promoter) and Oligo 3R(−) [25]. The resulting PCR product was linearized using the restriction site *Age*I immediately downstream the poly(A) tail and used as a template for in vitro transcription. [α-^32^P]UTP-labeled 6xB-3′UTR RNA was synthesized using the T7 MaxiScript in vitro transcription kit (Ambion) and purified by passing through the Mini Quick Spin RNA column (Roche) [25],[62]. To assay deadenylation, portions (8 µl) of nuclease-treated and supplemented Krebs extract, depleted of either hnRNP-Q2 or both hnRNP-Q2 and PABP, were mixed first with the indicated amounts of recombinant hnRNP-Q2, PABP, or Paip2 and then with 0.1 ng purified [α-^32^P]UTP-labeled 6xB-3′UTR RNA in a total volume of 10 µl. The reactions were incubated at 30°C for the indicated times, after which RNA was extracted with TRIzol reagent (Invitrogen) and analyzed by 4.5% polyacrylamide-urea gel electrophoresis. The dried gels were analyzed with a Typhoon PhosphorImager (GE Healthcare).

### Let-7a miRNA-Mediated Repression Analysis

Nearly confluent L929 cells, control and hnRNP-Q2 knockdown, were transfected with 100 ng of pRL or pRL-6xB and 50 ng of pFL in six-well plates using Lipofectamine 2000 (Invitrogen) [69]. Where indicated, 2′-*O*-Me antisense oligonucleotides complementary to either Let-7a or miR-122a miRNA (Dharmacon) were co-transfected at a final concentration of 90 nM [69]. Cells were split after 24 h. RL and FL activities were measured 48 h posttransfection using Dual-Luciferase Assay Kit (Promega) and their ratio was determined. The results from three estimations are represented as means ± SD. To analyze the levels of RL and RL-6xB mRNAs, RNA was extracted from a spare set of transfected cells by TRIzol and subjected to Northern blotting as described previously [69],[90]. The levels of RL and RL-6xB mRNAs were normalized against a control FL mRNA.

### Statistical Analysis

Statistical significance of the differences between means was evaluated using a Student's paired *t* test with a two-tailed distribution. The differences were considered significant at *p*<0.05.

## Supporting Information

Figure S1 Identification of p68. (A) Poly(A) interacting HeLa cytoplasmic proteins. HeLa S10 extract was depleted of PABP using GST-Paip2 affinity matrix and incubated with poly(A) Sepharose at 4°C for 1 h while mixing on a rotator. The beads were washed three times with 0.2 M KCl in buffer B (20 mM Tris-HCl, pH 7.5, 1 mM MgCl_2_, and 1 mM DTT). The poly(A) interacting proteins were sequentially eluted from the beads with 1 M KCl and 2 M LiCl in buffer B, concentrated, and analyzed by SDS-PAGE and Coomassie blue R-250 staining. Two distinct bands (1 and 2) of 1 M KCl eluate in the 70 K area of the gel were excised and analyzed by mass spectrometry. (B) Immunoprecipitation of p68. PABP-depleted RRL was subjected to UV-induced crosslinking with the ^32^P-poly(A) tail of globin mRNA. The labeled proteins were immunoprecipitated with anti-PABP rabbit polyclonal antibody, anti-hnRNP-Q mouse monoclonal antibody (18E4), anti-hsp70 rabbit polyclonal antibody, or anti-hsp70 mouse monoclonal antibody, as indicated. Precipitated proteins were resolved by SDS-PAGE and detected by autoradiography. Crosslinking of control RRL and the positions of molecular mass markers are also shown. (C) Comparative Western blotting of RRL, Krebs, and HeLa S10 extracts (5 µl) using 18E4 anti-hnRNP-Q antibody. The positions of hnRNP-Q2 and molecular mass markers are indicated. Molecular mass similarity of some hnRNP-Q isoforms in HeLa cells did not permit their satisfactory resolution.
(TIF)

Figure S2 Subcellular distribution of hnRNP-Q. A HeLa cytoplasmic extract was fractionated by sucrose density gradient centrifugation. Optical density (Abs 254 nm) tracings of polysomes (top) and Western blot analyses of hnRNP-Q, PABP, Paip2, YB-1, eIF4A, eIF4E, and 40S ribosomal protein S6 in aliquots of the indicated fractions (bottom) are shown. The appearance of protein S6 in fractions 5 and 6 confirms the identity of the 40S peak.
(TIF)

Figure S3 Abundance of hnRNP-Q2 in translation mixtures. (A) Quantification of hnRNP-Q2 in a Krebs translation mixture. Five and 10 µl aliquots of the complete translation mixture (containing 2.5 µl and 5 µl of a Krebs S10 extract, respectively) were analyzed side by side with the indicated amounts of recombinant hnRNP-Q2. Roughly equal signals were generated by 5 µl of the translation mixture and 150 ng hnRNP-Q2, setting a value for hnRNP-Q2 concentration of ∼30 µg/ml (480 nM). (B) Quantification of hnRNP-Q2 in a RRL translation mixture performed as in (A). Five µl of the complete translation mixture (an equivalent of 3.5 µl of RRL) and 75 ng hnRNP-Q2 generated signals of similar intensity, setting a value for hnRNP-Q2 concentration of ∼15 µg/ml (240 nM).
(TIF)

Figure S4 Cap- and poly(A)-tail dependence of translation in nuclease-treated RRL as affected by potassium ion and mRNA concentrations. The indicated concentrations of capped or uncapped firefly luciferase mRNA, with or without the poly(A) tail (A_98_), were translated in RRL that was not supplemented (A, C) or supplemented (B, D) with 60 mM KCl. Incubation was at 32°C for 1 h. Luciferase activity in 1-µl aliquots of the samples and the stimulation of translation by mRNA capping (A, B) and poly(A) tailing (C and D) are presented.
(TIF)

Figure S5 80S initiation complex formation in Krebs extracts as affected by hnRNP-Q2. 80S ribosome binding to 3′ end labeled globin mRNA was performed in the normal (A) or hnRNP-Q2-depleted (B) Krebs extract in the presence of cycloheximide (0.6 mM). HnRNP-Q2 (15 µg/ml; triangles) or control buffer (squares) were added to the reaction mixtures where indicated. 80S initiation complex formation was analyzed as described for Figure 5A, B.
(TIF)

Figure S6 The eIF4-group initiation factors relieve hnRNP-Q2 mediated inhibition of translation. Cap-Luc-A_98_ mRNA was translated in hnRNP-Q2-depleted Krebs extract in the presence of control buffer or recombinant hnRNP-Q2 (15 µg/ml). Where indicated, eIF4F, eIF4A, eIF4E, and eIF4B were included in the reaction mixtures at 90, 60, 10, and 75 µg/ml concentrations, respectively. Inhibition of translation by hnRNP-Q2 is shown on the top.
(TIF)

Figure S7 Let-7a miRNA-dependent deadenylation of 6xB-3′UTR RNA in Krebs extracts. Kinetics of deadenylation of 6xB-3′UTR RNA in a nuclease-treated Krebs extract as affected by 10 nM anti-let-7a 2′-*O*-Me (Anti-let-7a) or the mutations in nucleotides complementary to the let-7a “seed” sequence in the 3′UTR (6xBMut-3′UTR). The positions of polyadenylated (A_98_) and deadenylated (A_0_) RNAs are indicated on the right. The data are the representative of three independent experiments.
(TIF)

Table S1 Identification of a protein from band 1 (Figure S1A) as hnRNP-Q by HPLC/nanospray tandem mass spectrometry of tryptic peptides.
(DOC)

## Abbreviations

HCV: hepatitis C virus
hnRNP-Q: heterogeneous nuclear ribonucleoprotein Q
IRES: internal ribosome entry site
PABP: the poly(A) binding protein
Paip2: PABP interacting protein 2
PV: poliovirus
RRL: rabbit reticulocyte lysate
SYNCRIP: synaptotagmin-binding, cytoplasmic RNA-interacting protein
