# Supplementary figures and images for: Control of Translation and miRNA-Dependent Repression by a Novel Poly(A) Binding Protein, hnRNP-Q
Source: PLoS Biol. 2013 May 21;11(5):e1001564. doi: 10.1371/journal.pbio.1001564 (PMC3660254; doi:10.1371/journal.pbio.1001564)

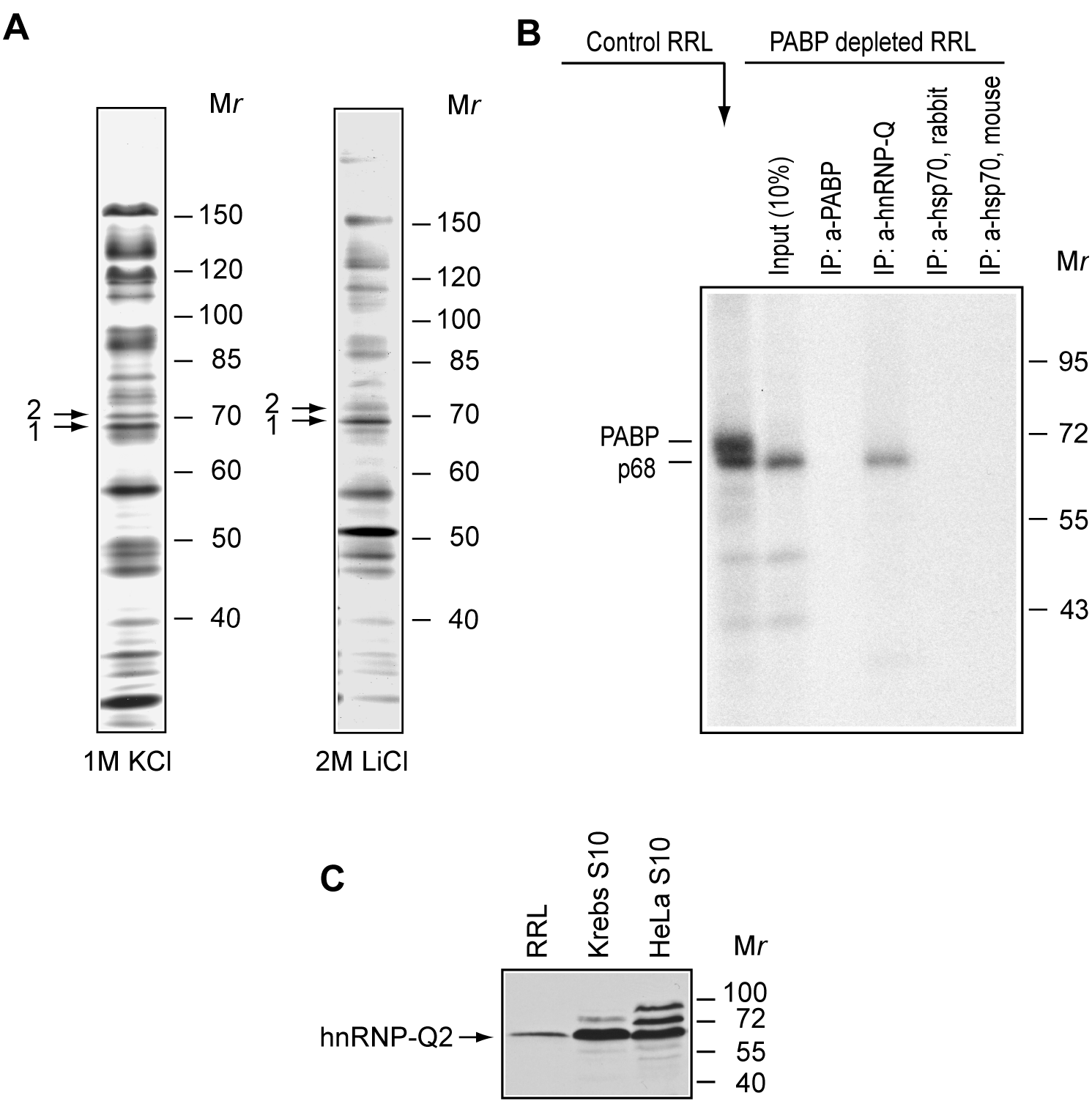

Supplement: Figure S1 — Identification of p68. (A) Poly(A) interacting HeLa cytoplasmic proteins. HeLa S10 extract was depleted of PABP using GST-Paip2 affinity matrix and incubated with poly(A) Sepharose at 4°C for 1 h while mixing on a rotator. The beads were washed three times with 0.2 M KCl in buffer B (20 mM Tris-HCl, pH 7.5, 1 mM MgCl2, and 1 mM DTT). The poly(A) interacting proteins were sequentially eluted from the beads with 1 M KCl and 2 M LiCl in buffer B, concentrated, and analyzed by SDS-PAGE and Coomassie blue R-250 staining. Two distinct bands (1 and 2) of 1 M KCl eluate in the 70 K area of the gel were excised and analyzed by mass spectrometry. (B) Immunoprecipitation of p68. PABP-depleted RRL was subjected to UV-induced crosslinking with the 32P-poly(A) tail of globin mRNA. The labeled proteins were immunoprecipitated with anti-PABP rabbit polyclonal antibody, anti-hnRNP-Q mouse monoclonal antibody (18E4), anti-hsp70 rabbit polyclonal antibody, or anti-hsp70 mouse monoclonal antibody, as indicated. Precipitated proteins were resolved by SDS-PAGE and detected by autoradiography. Crosslinking of control RRL and the positions of molecular mass markers are also shown. (C) Comparative Western blotting of RRL, Krebs, and HeLa S10 extracts (5 µl) using 18E4 anti-hnRNP-Q antibody. The positions of hnRNP-Q2 and molecular mass markers are indicated. Molecular mass similarity of some hnRNP-Q isoforms in HeLa cells did not permit their satisfactory resolution. (TIF) [file pbio.1001564.s001.tif]

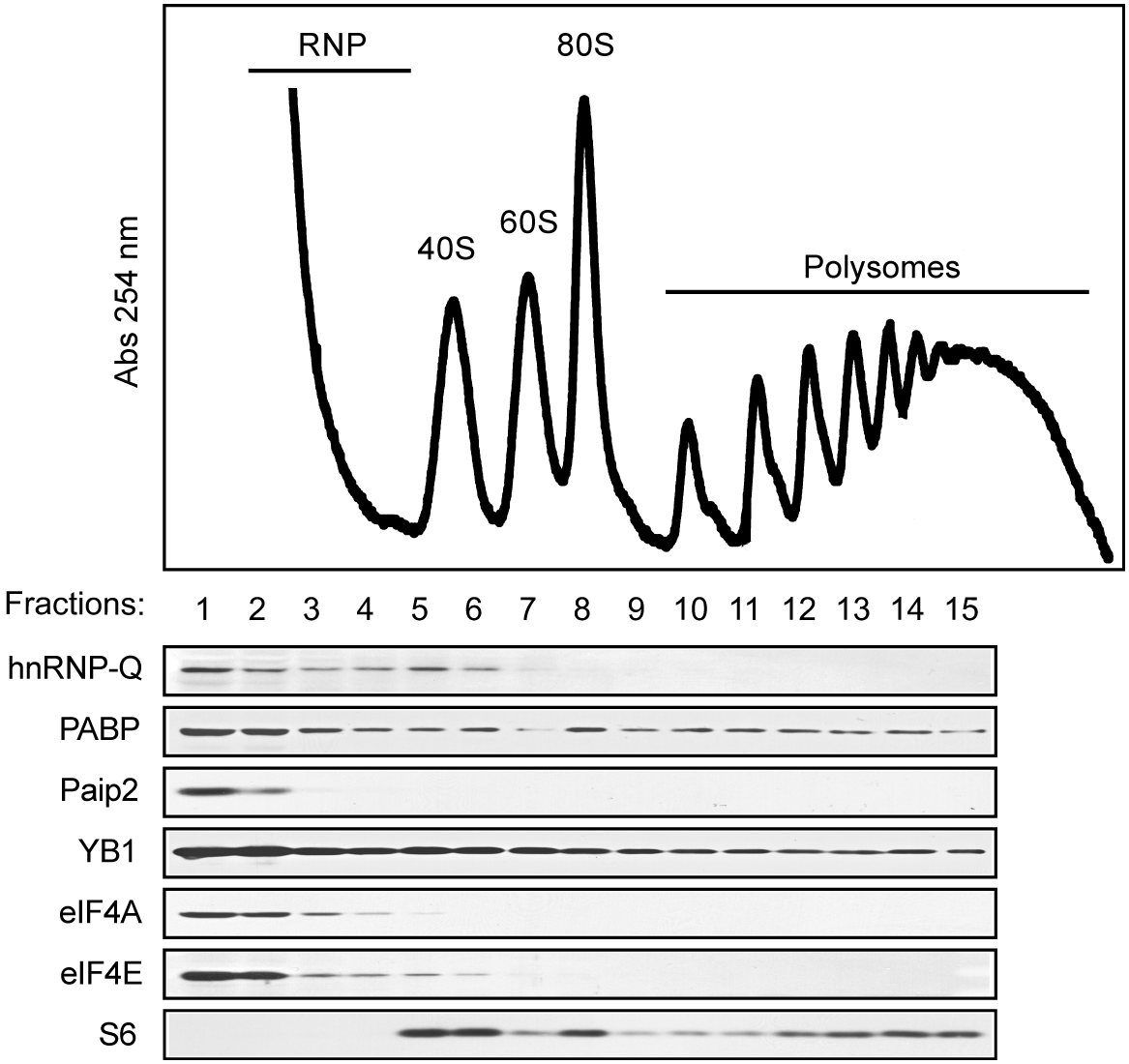

Supplement: Figure S2 — Subcellular distribution of hnRNP-Q. A HeLa cytoplasmic extract was fractionated by sucrose density gradient centrifugation. Optical density (Abs 254 nm) tracings of polysomes (top) and Western blot analyses of hnRNP-Q, PABP, Paip2, YB-1, eIF4A, eIF4E, and 40S ribosomal protein S6 in aliquots of the indicated fractions (bottom) are shown. The appearance of protein S6 in fractions 5 and 6 confirms the identity of the 40S peak. (TIF) [file pbio.1001564.s002.tif]

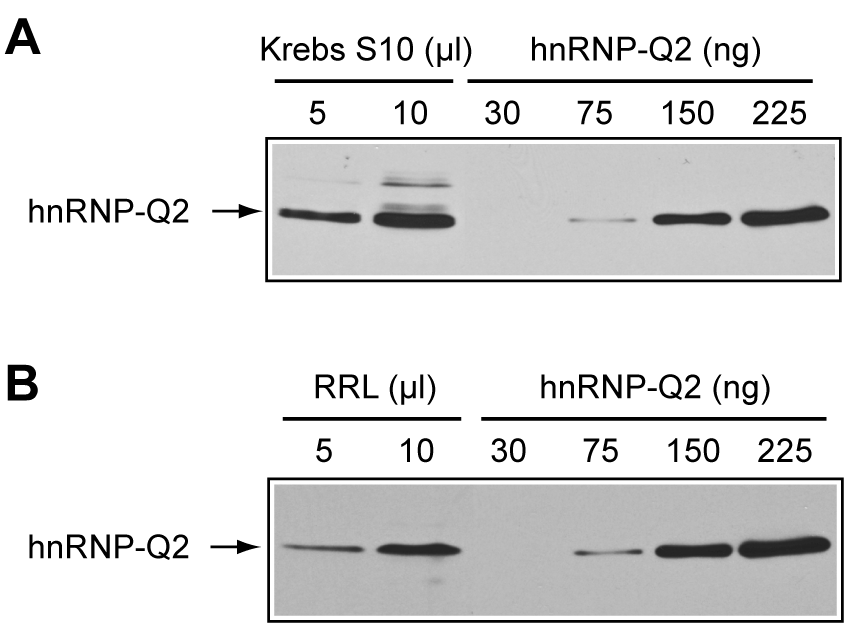

Supplement: Figure S3 — Abundance of hnRNP-Q2 in translation mixtures. (A) Quantification of hnRNP-Q2 in a Krebs translation mixture. Five and 10 µl aliquots of the complete translation mixture (containing 2.5 µl and 5 µl of a Krebs S10 extract, respectively) were analyzed side by side with the indicated amounts of recombinant hnRNP-Q2. Roughly equal signals were generated by 5 µl of the translation mixture and 150 ng hnRNP-Q2, setting a value for hnRNP-Q2 concentration of ∼30 µg/ml (480 nM). (B) Quantification of hnRNP-Q2 in a RRL translation mixture performed as in (A). Five µl of the complete translation mixture (an equivalent of 3.5 µl of RRL) and 75 ng hnRNP-Q2 generated signals of similar intensity, setting a value for hnRNP-Q2 concentration of ∼15 µg/ml (240 nM). (TIF) [file pbio.1001564.s003.tif]

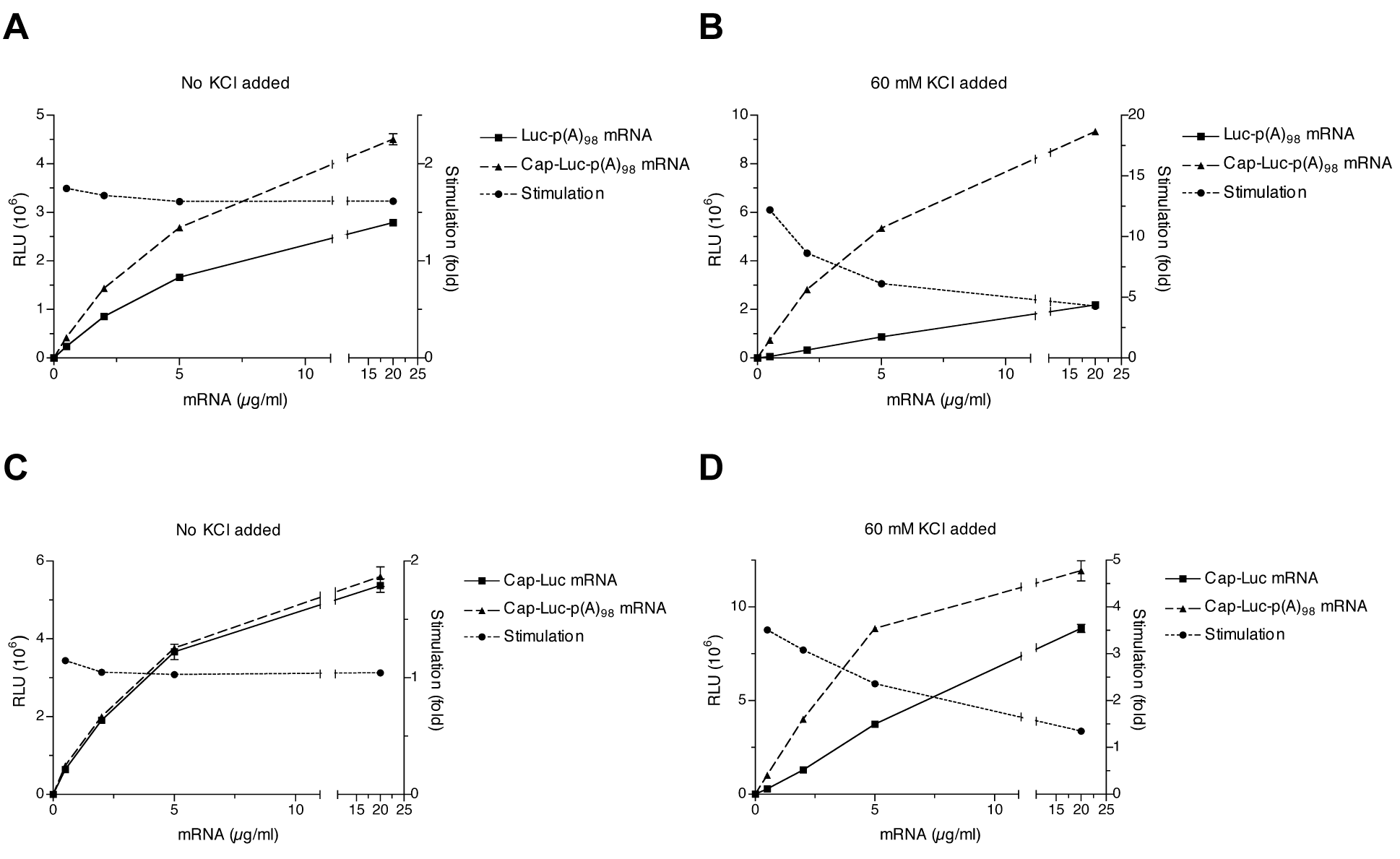

Supplement: Figure S4 — Cap- and poly(A)-tail dependence of translation in nuclease-treated RRL as affected by potassium ion and mRNA concentrations. The indicated concentrations of capped or uncapped firefly luciferase mRNA, with or without the poly(A) tail (A98), were translated in RRL that was not supplemented (A, C) or supplemented (B, D) with 60 mM KCl. Incubation was at 32°C for 1 h. Luciferase activity in 1-µl aliquots of the samples and the stimulation of translation by mRNA capping (A, B) and poly(A) tailing (C and D) are presented. (TIF) [file pbio.1001564.s004.tif]

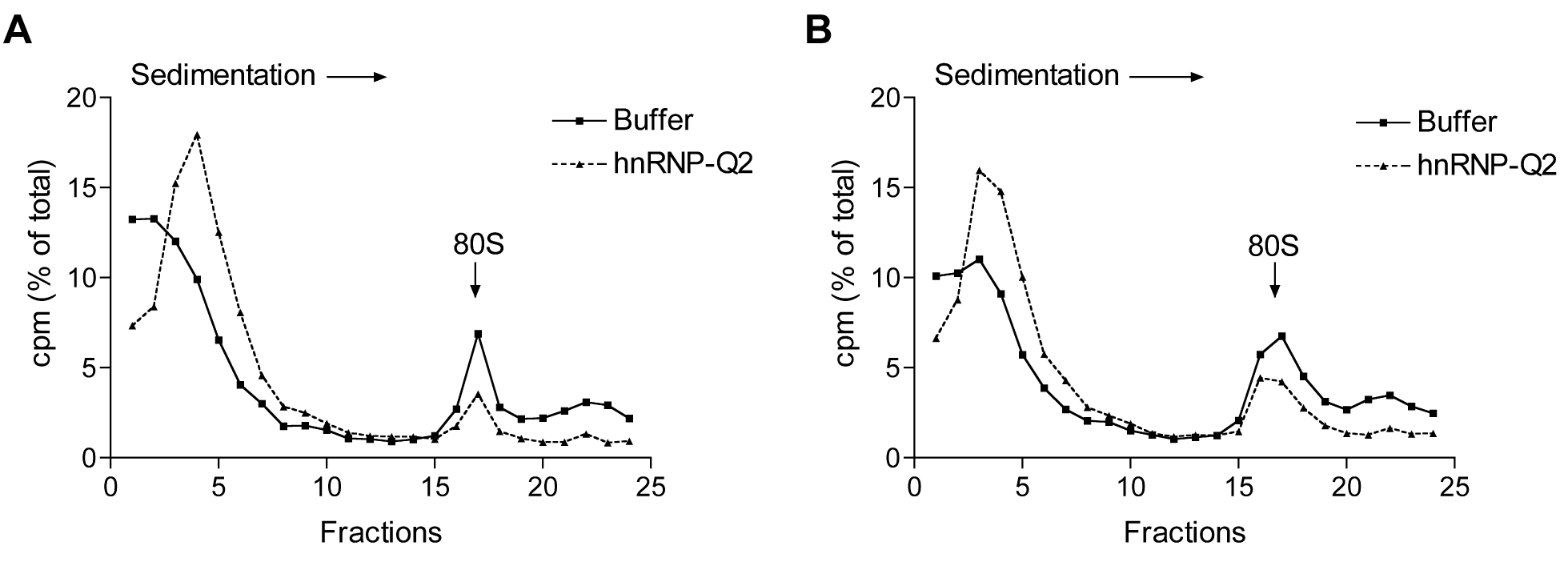

Supplement: Figure S5 — 80S initiation complex formation in Krebs extracts as affected by hnRNP-Q2. 80S ribosome binding to 3′ end labeled globin mRNA was performed in the normal (A) or hnRNP-Q2-depleted (B) Krebs extract in the presence of cycloheximide (0.6 mM). HnRNP-Q2 (15 µg/ml; triangles) or control buffer (squares) were added to the reaction mixtures where indicated. 80S initiation complex formation was analyzed as described for Figure 5A, B. (TIF) [file pbio.1001564.s005.tif]

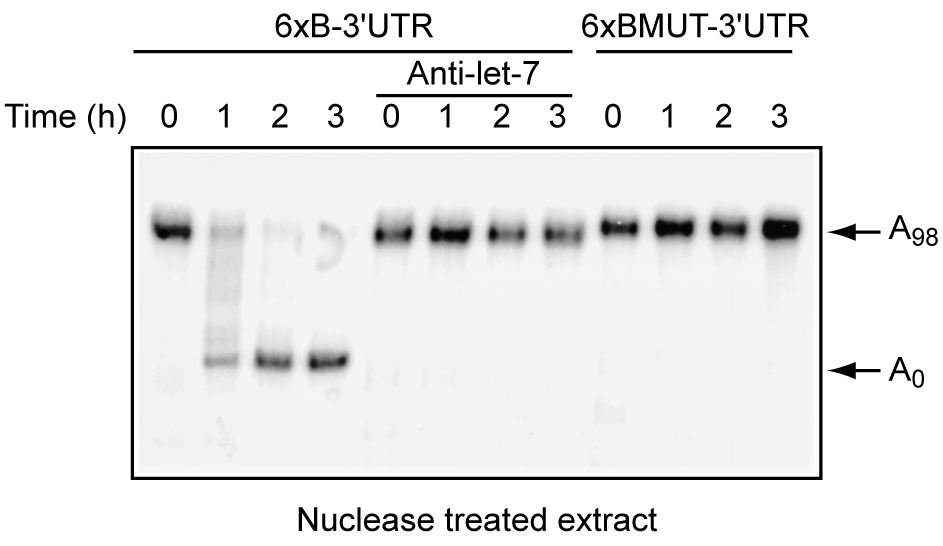

Supplement: Figure S7 — Let-7a miRNA-dependent deadenylation of 6xB-3′UTR RNA in Krebs extracts. Kinetics of deadenylation of 6xB-3′UTR RNA in a nuclease-treated Krebs extract as affected by 10 nM anti-let-7a 2′-O-Me (Anti-let-7a) or the mutations in nucleotides complementary to the let-7a “seed” sequence in the 3′UTR (6xBMut-3′UTR). The positions of polyadenylated (A98) and deadenylated (A0) RNAs are indicated on the right. The data are the representative of three independent experiments. (TIF) [file pbio.1001564.s007.tif]
