# Supplementary material for: Control of Translation and miRNA-Dependent Repression by a Novel Poly(A) Binding Protein, hnRNP-Q
Source: PLoS Biol. 2013 May 21;11(5):e1001564. doi: 10.1371/journal.pbio.1001564 (PMC3660254; doi:10.1371/journal.pbio.1001564)
Supplement: Table S1 — Identification of a protein from band 1 (Figure S1A) as hnRNP-Q by HPLC/nanospray tandem mass spectrometry of tryptic peptides. (DOC) [file pbio.1001564.s008.doc]

**Table S1.** Identification of a protein from band 1 (Figure S1A) as hnRNP-Q by HPLC/nanospray tandem mass-spectrometry of tryptic peptides

| **Peptide sequence** | **Amino acid residues*** |
| --- | --- |
| YGGPPPDSVYSGQQPSVGTEIFVGK | 144-168 |
| VTEGLTDVILYHQPDDK | 266-282 |
| VAEKLDEIYVAGLVAHSDLDER | 39-60 |
| TKEQILEEFSK | 255-265 |
| TGYTLDVTTGQR | 131-142 |
| SGKHIGVCISVANNR | 230-244 |
| SAFLCGVMK | 92-100 |
| NLANTVTEEILEK | 344-356 |
| LMMDPLTGLNR | 193-203 |
| LKDYAFIHFDER | 370-381 |
| LDEIYVAGLVAHSDLDER | 43-60 |
| KYGGPPPDSVYSGQQPSVGTEIFVGK | 143-168 |
| HIGVCISVANNR | 233-244 |
| GYAFVTFCTK | 204-213 |
| EQILEEFSK | 257-265 |
| EFNEDGALAVLQQFK | 67-81 |
| DYAFIHFDER | 372-381 |
| DLFEDELVPLFEK | 172-184 |
| DLEGENIEIVFAKPPDQK | 395-412 |
| AMEEMNGKDLEGENIEIVFAKPPDQK | 387-412 |
| AIEALKEFNEDGALAVLQQFK | 61-81 |
| AGPIWDLR | 185-192 |

* Amino acid residues of unique peptides are numbered according to the sequence of human hnRNP-Q (UniProtKB accession number: O60506)
